# Prioritisation of Compounds for 3CL^pro^ Inhibitor Development on SARS-CoV-2 Variants

**DOI:** 10.3390/molecules26103003

**Published:** 2021-05-18

**Authors:** Marko Jukič, Blaž Škrlj, Gašper Tomšič, Sebastian Pleško, Črtomir Podlipnik, Urban Bren

**Affiliations:** 1 Laboratory of Physical Chemistry and Chemical Thermodynamics, Faculty of Chemistry and Chemical Engineering, University of Maribor, Smetanova ulica 17, SI-2000 Maribor, Slovenia; marko.jukic@um.si; 2 Faculty of Mathematics, Natural Sciences and Information Technologies, University of Primorska, Glagoljaška 8, SI-6000 Koper, Slovenia; 3 Institute Jožef Stefan, Jamova cesta 39, SI-1000 Ljubljana, Slovenia; blaz.skrlj@ijs.si; 4 Independent Researcher, Cesta Cirila Kosmača 66, SI-1000 Ljubljana, Slovenia; gasper.tomsic@icloud.com; 5 Erudio, Litostrojska Cesta 40, SI-1000 Ljubljana, Slovenia; sebastian.plesko@erudio.si; 6 Faculty of Chemistry and Chemical Technology, University of Ljubljana, Večna pot 113, SI-1000 Ljubljana, Slovenia

**Keywords:** COVID-19, SARS-CoV-2, M^pro^, 3CL^pro^, 3C-like protease, high-throughput, virtual screening, inhibitors, in silico drug design, chemical library design, machine learning, compound prioritisation

## Abstract

COVID-19 represents a new potentially life-threatening illness caused by severe acute respiratory syndrome coronavirus 2 or SARS-CoV-2 pathogen. In 2021, new variants of the virus with multiple key mutations have emerged, such as B.1.1.7, B.1.351, P.1 and B.1.617, and are threatening to render available vaccines or potential drugs ineffective. In this regard, we highlight 3CL^pro^, the main viral protease, as a valuable therapeutic target that possesses no mutations in the described pandemically relevant variants. 3CL^pro^ could therefore provide trans-variant effectiveness that is supported by structural studies and possesses readily available biological evaluation experiments. With this in mind, we performed a high throughput virtual screening experiment using CmDock and the “*In-Stock*” chemical library to prepare prioritisation lists of compounds for further studies. We coupled the virtual screening experiment to a machine learning-supported classification and activity regression study to bring maximal enrichment and available structural data on known 3CL^pro^ inhibitors to the prepared focused libraries. All virtual screening hits are classified according to 3CL^pro^ inhibitor, viral cysteine protease or remaining chemical space based on the calculated set of 208 chemical descriptors. Last but not least, we analysed if the current set of 3CL^pro^ inhibitors could be used in activity prediction and observed that the field of 3CL^pro^ inhibitors is drastically under-represented compared to the chemical space of viral cysteine protease inhibitors. We postulate that this methodology of 3CL^pro^ inhibitor library preparation and compound prioritisation far surpass the selection of compounds from available commercial “corona focused libraries”.

## 1. Introduction

Coronavirus disease (COVID-19) is an infectious disease caused by a novel severe acute respiratory syndrome coronavirus 2 or SARS-CoV-2. COVID-19 was initially reported in Wuhan province in China and was declared as a global pandemic [1]. COVID-19 is a severe illness similar to flu, with major symptoms being cough, fever and breathing difficulty. Furthermore, the illness can cause systemic inflammation [2,3]. The pathogen SARS-CoV-2 belongs to the *Coronaviridae* family, an enveloped positive-sense single-stranded (+ssRNA) RNA virus, and is closely related to the previously described SARS-CoV and MERS-CoV coronaviruses [4]. The SARS-CoV-2 genome shares 82% sequence identity with SARS-CoV and 90% identity with MERS-CoV and shares common pathogenesis mechanisms [5].

Currently, there are registered vaccines available to fight this global crisis, and multiple vaccine development programs are underway [6,7,8,9]. However, there are only a handful of therapeutic options for COVID-19 treatment and no registered antiviral drugs against SARS-CoV-2 at the moment [10,11,12,13,14]. Furthermore, research suggests a minimal variation in the genome sequence of SARS-CoV-2 pathogen may translate to changes in the structures of viral proteins rendering available vaccines or even medicines ineffective [15]. In late 2020, early 2021, the emergence of the new SARS-CoV-2 variants was reported; namely the B.1.1.7 variant, dubbed the UK variant, the B.1.351 variant or South African variant and B.1.617, known as the Indian variant [16,17,18]. Both variants are reported to possess N501Y mutation in the RBD (receptor binding domain) of the Sprot (spike protein) that is associated with increased viral transmission [19]. The South African variant also possesses K417N and E484K mutations in the Sprot that are potentially responsible for the diminished binding of viral Sprot to host antibodies [20]. In Brazil, the P.1 variant with known N501Y, E484K and novel K417T mutation at the Sprot was identified [21]. A SARS-CoV-2 variant summary is presented in Table 1.

It is of key interest that no mutations have been observed on SARS-CoV-2 3CL^pro^ main protease (location 3292 → 3582 on ORF1ab polyprotein; YP_009724389.1) and only one mutation on SARS-CoV-2 PL^pro^ protease (location 1564 → 1882 on ORF1ab polyprotein; YP_009724389.1) for each variant [22]. It should be stressed, however, that this does not mean that these proteins cannot mutate in the future. In this context, 3CL^pro^ is an attractive target for novel antiviral discovery with a potential for trans-variant activity [23,24,25,26]

**Figure 1:**
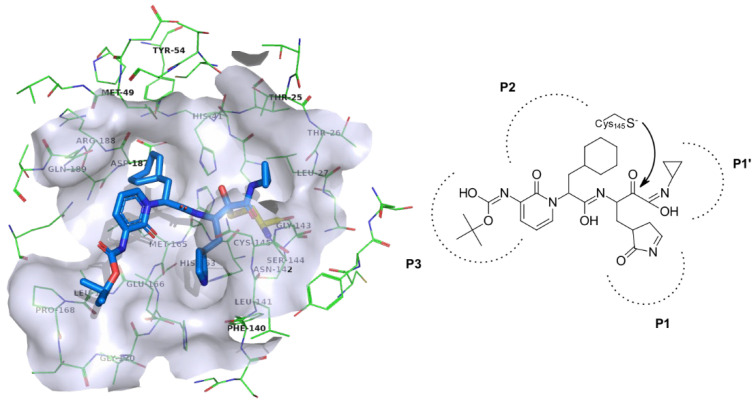
Active site of SARS-CoV-2 3Cl^pro^ or M^pro^ enzyme (PDB ID: 6Y7M) with emphasised small molecule and pocket designation on the right. Active site residues are depicted in green coloured line model with emphasized transparent blue-white surface 6 Å around the small molecule inhibitor depicted in blue coloured stick model.

3CL^pro^ (Picornain 3C-like protease, also referred to as M^pro^ for main protease) is a homodimeric cysteine protease (EC 3.4.22.69) and is 96% sequence identical with the SARS-CoV M^pro^ [27]. The enzyme belongs to the family C30 peptidases in the PA peptidases clan. It consists of two 306 residues long polypeptide chains, which fold into three domains (I, II and III). Domains I and II have an antiparallel β-barrel structure, while domain III is composed of 5 α-helices, which connect to domain II by a long loop region. Judging by its dimer interface, it seems the dimer (comprised of protomers A and B) is an active form which is considerably less efficient when isolated in its monomer form. The active site is readily accessible to the solvent and is located distal to the dimer interface [28]. The substrate binding site is comprised of pockets P1, P1’, P2 and P3. The P1 subsite is formed with Phe140, Asn142, Glu166, His163 and His172 residues (Figure 1) and two conserved water molecules. P2 is a deep pocket comprised of His41, Met49, Tyr54, Met165 and Asp187 residues while P3 is defined by Leu168 and flanked by Glu166, Pro168 and Gly170 [29]. Proteolysis occurs via a catalytic dyad defined by Cys145 and His41 [30]. The enzyme is responsible for cleavage on no less than 11 sites on the large viral polyprotein 1ab. Cleavage generally follows the pattern Leu/Phe/Met-Gln ↓ Gly/Ser/Ala (↓ denotes the cleavage site). Glutamine at the P1 position is crucial for proteolysis to occur. As there are no known native human enzymes with such cleavage sites, M^pro^ looks to be an ideal drug target, since there is a low risk for toxic effects on host cells [31,32,33]. Structural data on the enzyme is available and reporter assays developed making this target suitable for novel antiviral design [34,35].

In this study, we perform a high-throughput virtual screening (HTVS) experiment coupled with machine learning classification to offer a prioritisation approach for compounds with potential activity on SARS-CoV-2 3CL^pro^. We employ fast methodologies to cover a comprehensive chemical space based on molecular docking scores in order to offer prioritisation lists of compounds for further free energy calculations and suitable for biological evaluation [36]. We focus on identifying novel potential non-covalent protease inhibitors, as we firmly believe they offer the flexibility of optimisation and synthetic or commercial availability [37,38].

## 2. Results and Discussion

### 2.1. Database Preparation

**Figure 2:**
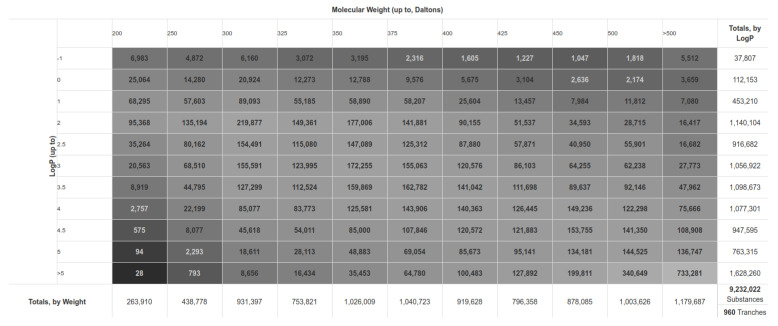
Detailed ZINC 15 database “*In-Stock*” subset tranche description with a total of 9,322,002 compounds used for further calculations (As obtained from the https://zinc15.docking.org; accessed on 8 May 2021; at the time of smiles compound download; the database on the master server is continuously updating).

For our HTVS (high-throughput virtual screening) experiment, we employed the ZINC 15 library [39]. To produce robust results that would enable downstream experimental support and biological evaluation, we specifically selected the "*In-Stock*" library subset that includes commercially readily-available compounds. The subset was trenched to exclude small fragments below the molecular weight of 200 g/mol and included 9,232,022 compounds in total (Figure 2). The next step was compound ionisation at the pH of 7.4, the calculation of initial 3D conformations for the whole database, the enumeration of undefined chiral centres, and the removal of structural faults. Smiles strings were syntactically validated (all rings/branches closed, no illegal atom types) with RDKit software (MolFromSmiles). Ionisation was performed using OpenEye QUACPAC 2.1.1.0 software (OpenEye Scientific Software, Inc., Santa Fe, NM, USA; www.eyesopen.com; accessed on 8 May 2021) with FixpKa module and ionise 7.4 parameters. The initial 3D conformation was calculated with OpenEye OMEGA 2.5.1.4 software (OpenEye Scientific Software, Inc., Santa Fe, NM, USA; www.eyesopen.com). The following parameters were used: maxconfs 1 and flipper: true [40].

### 2.2. Binding Site Identification

**Figure 3:**
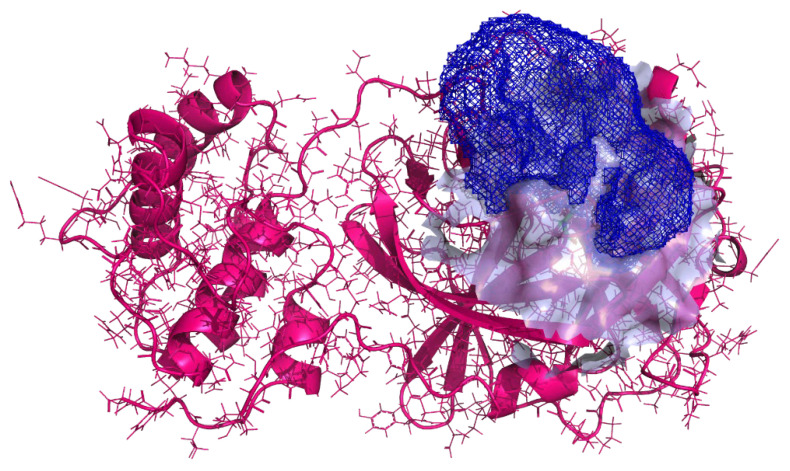
Generated receptor volume with the 3CL^pro^ PDB ID: 6Y7M, the active site near the Cys145 residue and the docking volume defined as a sphere of 7 Å around the reference ligand OEW. Protein is depicted as a pink-magenta-coloured cartoon model with residues emphasised in the line model and coloured atoms (carbon in green, oxygen in red, nitrogen in blue and hydrogen in white colour) with a blue-white transparent active site surface. Docking volume boundary mesh is depicted in blue colour. Isomesh (0.99) was constructed using PyMol 2.1.0 using a grid calculated with cmgrid software (v 0.1.1).

Upon examination of available SARS-CoV-2 3CL^pro^ crystal structures at the time, we selected the complex (PDB ID: 6Y7M) with an excellent resolution of 1.9 Å deposited by Zhang L. et al. [41]. This complex contained a relatively large peptidomimetic inhibitor OEW (MW = 585.69 g/mol) occupying major pockets at the enzyme active site, essentially keeping the S1 pocket in the correct shape and the enzyme in the active conformation. After superimposition to a reference structure (PDB ID:6LU7) by Yang et al., the catalytic binding pocket was defined around Cys145 as published previously by Jukic et al. [29,42]. Finally, the target was prepared as a sphere of 7 Å around the ligand for docking volume calculation using the CmDock docking package (https://gitlab.com/Jukic/cmdock/; accessed on 8 May 2021; Figure 3; details are in Supplementary Materials).

### 2.3. HTVS

**Figure 4:**
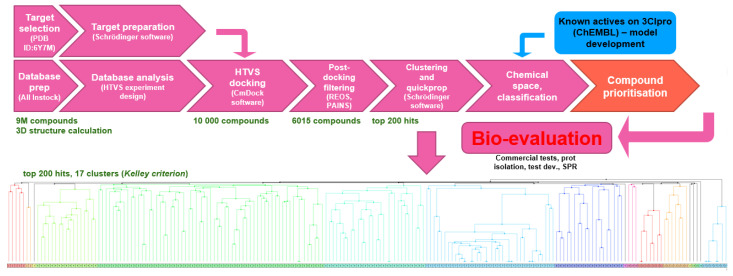
HTVS workflow with post-docking filtering, cluster analysis, and compound classification according to the chemical space of 3CL^pro^ inhibitors collected in the ChEMBL database.

For the HTVS campaign, the complete pre-prepared database was docked using CmDock into the prepared receptor binding site to afford 1‰ of top-scoring compounds with the Z-score cutoff of –7.7 (Figure 4).

**Figure 5:**
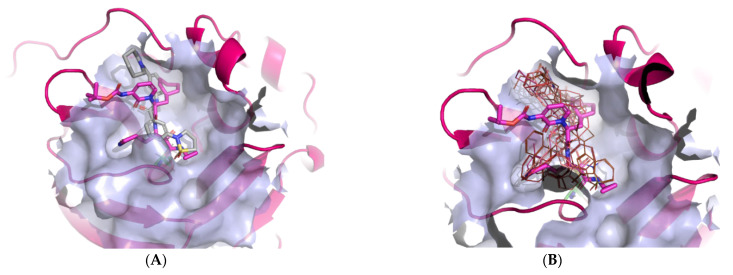
Calculated bound conformations in the 3CL^pro^ active site of the top-scoring hit compounds. Protein is presented in a cartoon model coloured pink with an emphasised molecular surface in light-blue colour. (**A**); the reference OEW ligand is presented in stick model cored magenta, while the top-scoring hit is coloured grey. (**B**) the reference OEW ligand is presented in stick model cored magenta while the top 10 scoring compounds are depicted in red-coloured line representations to emphasise their analogous binding mode in the P1-P2-P3 pockets of the active site.

In order to inspect all chemical space, filtering was done post-docking, where subsequently PAINS [43,44,45] and REOS structures were filtered out [46,47]. KNIME software with RDKit nodes was applied to compare all structures in the library to the selection of SMARTS-formatted PAINS and to remove flagged hits from the database followed by REOS filtering with Schrödinger SMD software (Schrödinger LLC., New York, NY, USA). In this way, approximately 40% of the pan-assay interfering and reactive compounds (with labile functional groups) were filtered out, and the top 200 scoring compounds were examined by cluster analysis and classified in the current 3CL^pro^-actives space. Hierarchical clustering was performed using Schrödinger SMD (Schrödinger LLC., New York, NY, USA) using Molprint2D hashed fingerprints, Tanimoto similarity metric and average cluster linkage method; the 17 clusters have been estimated by the Kelley criterion [48]. The final compound selection was performed with top-scoring hits with consideration of QuickProp QPlogS (Schrödinger LLC., New York, NY, USA) descriptor (>−6.5) in order to focus on compounds that have a greater potential to be soluble. Complete QuickProp descriptor set was calculated to flag all un-/desired properties and enable further custom prioritisation of compounds, and the whole set of top 200 hits is supplied in the Supplementary Materials for the convenience of future research. Upon examination of all top-scoring compounds, we identified that they all conform to the classic P1-P2-P3 binding pockets, as described previously [42]. Predicted bound conformations for the top-scoring hit as well as for the first ten hit compounds are analogous (and in accordance to the crystal ligand OEW), and compounds interact with key Thr25, Leu27, Gly143, Ser144, Cys145, His163, His164, Met165, Glu166, Asp187, Thr190, Gln189 and Gln192 residues at the 3CL^pro^ active site (Table 2, Figure 5).

### 2.4. Chemical Space Prediction and Classification Supported by Machine Learning

We also investigated whether state-of-the-art machine learning algorithms could be of use for compound prioritisation. In this work, we implemented four different machine learning algorithms capable of detecting various complexities and evaluated their performance on the training data. The training data for the regression experiment consisted of all compounds with activity on the 3CL^pro^ enzyme (ChEMBL standard value IC50 in nM, 133 compounds with 1k decoy set, available in Supplementary Materials) present in the ChEMBL database, while the training data for classification consisted of chemical libraries active on 3CL^pro^ as well as actives on all viral cysteine proteases (3145 compounds with 1k decoy set, available in Supplementary Materials) [49]. For all compounds, we used RDKit.Chem package. RDKit.Chem.Descriptors module calculated a set of 208 chemical descriptors in order to capture the respective chemical space. In this manner, we could effectively classify compounds in the context of their representative chemical space: similar to known 3CL^pro^ inhibitors, similar to known viral cysteine protease inhibitors, or belonging to a different chemical space to help in future design directions for individual scaffolds. The learning algorithms were (from simplest to more complex ones): a majority classifier (prediction of the most common class), a logistic regression classifier (linear classifier), and two non-linear classifiers, namely a deep feedforward neural network, designed specifically for this task, as well as the extreme gradient boosting machines (XGB), which are considered a strong learner in contemporary chemoinformatics [50,51,52,53].

## 3. Materials and Methods

### 3.1. Target Preparation

Complex (PDB ID: 6Y7M) contains {tert}-butyl ~{N}-[1-[(2~{S})-3-cyclohexyl-1-[[(2~{S},3~{R})-4-(cyclopropylamino)-3-oxidanyl-4-oxidanylidene-1-[(3~{R})-2-oxidanylidene-3,4-dihydropyrrol-3-yl]butan-2-yl]amino]-1-oxidanylidene-propan-2-yl]-2-oxidanylidene-pyridin-3-yl]carbamate (OEW), a peptide-like covalent inhibitor where thiohemiketal is formed by the nucleophilic attack of the catalytic cysteine (Cys145) onto the carbonyl group of the inhibitor. Residue conformation at the active site was checked by superposition with PDB ID: 6M2N and 6XMK with an all atom RMSD of 0.330 and 0.662 Å, respectively [54,55]. For the docking receptor preparation, the covalent bond was cleaved, a small molecule removed, and the Cys145 amino-acid residue regenerated (Open Source PyMOL, release 2.1). The target was prepared using Schrödinger Small-Molecule Discovery Suite (Schrödinger LLC., New York), protein preparation module. Missing hydrogens were added, h-bonding network was optimised using PROPKA tool at pH 7.4, waters were removed, and restrained minimisation was performed with a convergence of heavy atoms towards 0.3 Å. Finally, using the cmcavity program from CmDock docking package (https://gitlab.com/Jukic/cmdock/; accessed on 8 May 2021), we generated a docking receptor [56,57,58]. The reference ligand method was employed for cavity calculation (*receptor* definition), where we used the OEW-cleaved regenerated ligand as a reference and a sphere of 7 Å around the ligand for the docking volume (cavity volume) calculation. We calculated a total cavity volume of 3106.25 A^3^ and included the calculated cavity (Cavity #1) in the *docking receptor* definition. Cavity #1 parameters were the size of 24850 points; min = (−33.5,−53.5,−8.5); max = (−13,−26.5,12); centre = (−24.4138,−38.9632,−0.179235); coordinates and extent = (20.5,27,20.5) Å (Figure 3, details are in Supplementary Materials).

### 3.2. Virtual Screening Experiment Design

For the virtual screening experiment (HTVS), a docking approach with a robust CmDock software, the prepared compound database, and the docking receptor (Cavity #1), as described in the previous section, were employed [56,57]. Firstly, we conducted a redocking experiment. The reference-regenerated OEW peptidomimetic ligand (PDB ID: 6Y7M) was prepared as a SMILES string and energy-minimised in Ligprep tool from Schrödinger SMD using the OPLS 3e forcefield. The minimised structure was subsequently used as an input for the redocking experiment into the prepared receptor in a non-covalent manner. Applied parameters for the CmDock software (v 0.1.1) were standard docking protocol (dock.prm) with 100 runs, no constraints, and no score filters. We successfully retrieved the crystal-complex binding conformation of the OEW ligand with an RMSD of 1.34 Å. Furthermore, we calculated the receiver operating characteristic (ROC) curve to validate the performance of the classifier docking method. We selected a set of known SARS-CoV 3CL^pro^ inhibitors from the ChEMBL database with experimental IC_50_ < 100 μM values and created a testing database by the addition of negative control compounds that were calculated decoys based on employed actives using DUD-E: A Database of Useful (Docking) Decoys [59]. Upon using 1% and 10% of actives in the test database, we obtained a ROC AUC of 0.80 and 0.79, respectively. We also employed the activity data from the PostEra Covid Moonshot project (https://covid.postera.ai/covid/activity_data; accessed on 8 May 2021). We selected compounds with pIC_50_ above 7 as true actives and compounds with pIC_50_ up to 4.00436 as inactives or experimental decoys (compounds with no data were left out). When using 2% and 10% of actives in the test set, we obtained ROC AUCs of 0.61, indicating that our docking protocol can indeed identify active compounds and produce enriched libraries.

In order to effectively utilise CPU-time in downstream calculations, we analysed the chemical library performance in HTVS. We sampled a random 10% population of the designed library and performed an exhaustive docking experiment on 977,600 molecules (dock.prm protocol, 100 runs per molecule, no constraints, and no score filter). We then analysed the docking results using the sdreport script (part of CmDock package) and KNIME software, where the mean docking score was −12.317, and the standard deviation was 4.273. Upon passthrough analysis using rbhtfinder script (part of CmDock package, parameters: −15 and −20), the HTVS analysis afforded an optimal HTVS experiment workflow where up to 5 runs were performed for molecules that possessed docking score of −18 and up to 15 runs for molecules that were found with docking scores of −25. In the case of docking scores lower than −25, 50 runs were performed. The first filter was passed by 25.7% of molecules, the second filter was passed by 1.71% of molecules, and the run was on average 7.8% CPU-time compared to the exhaustive docking run-time, thereby effectively improving the HTVS efficiency.

### 3.3. Machine Learning

The deep neural network consisted of six hidden layers, where each layer was activated with the ReLU activation, followed by dropout-based regularisation, set to 0.3 [60]. The final layer of the neural network was activated with a softmax function to obtain a distribution across the space of possible classes. During prediction, the most probable class is selected (argmax across class probabilities). The extreme gradient boosting machines are a type of tree-based ensemble classifier capable of fast learning and robust and accurate predictions. The evaluation regime was designed as follows: we randomly sampled ten different stratified splits, where the training data was used to learn the models, followed by the evaluation of their predictions on the test data. The splits were in ratio 8:2. We report the average performance with a standard deviation for the macro F1 score (the task considered is a multiclass classification). Apart from the classification task, the point was to differentiate between chemical spaces occupied by known 3Clpro inhibitors reported in ChEMBL, known viral cysteine protease inhibitors in the ChEMBL database, and other chemical space. We also conducted a similar series of experiments to assess to what extent machine learning can predict the inhibition of 3CL^pro^ directly (on the basis of the ChEMBL standard value IC50 in nM), which is a regression task. The regression variants of the algorithms mentioned above were considered, namely the XGB with the “req:squarederror” loss, the neural network’s classification head was replaced with a regression one, and instead of logistic regression, we used a simple linear regressor. The neural network that performed best consisted of seven (regularized) hidden layers; other hyperparameters were the same as in the classification scenario. As the neural network’s expected output is a positive real number, the final activation used was a ReLU as well, as we observed faster convergence compared to using no activation at all. Mean squared errors with standard deviation across ten random splits (see the previous section) are reported (Table 3).

The neural network and XGB performed, as expected, the best, followed by a simpler linear learner. Compared to the majority baseline, e.g., the neural network’s performance is substantially higher, indicating that it learned to differentiate between the classes. On the contrary, the average-training baseline (averaged train predictions) performed better than the linear classifier, indicating that the regression problem at hand is relatively hard and potentially not linearly separable. The XGB and neural networks performed better than this baseline, albeit not by a large margin, indicating the actives 3CL^pro^ dataset was insufficient for effective training. However, the regression task similarly indicates that it is possible to use non-linear models for direct activity prediction necessitating good data collection on 3CL^pro^ actives in the future. Furthermore, the authors are aware of the generalisation of the idea on other well-represented datasets and postulate its great value in future in silico drug design.

## 4. Conclusions

Herein, we presented a novel in silico approach towards compound prioritisation in the design of novel 3CL^pro^ inhibitors. Rather than relying on commercial “corona-focused libraries”, we performed a robust and efficient HTVS experiment to obtain virtual hit compounds. We then coupled the method to a machine learning classification experiment where each compound is classified into the chemical space of 3CL^pro^ inhibitors, into general viral cysteine protease inhibitors, or into a completely novel unrelated chemical space. In this way, medicinal chemists can turn their attention towards compounds that would otherwise escape and derive insight into the possible mechanism of action. Therefore, we employed a complete library of 3CL^pro^ inhibitors and viral cysteine protease inhibitors obtained from the ChEMBL library using the calculated ensemble of 208 descriptors for each compound in the library. We reported ten top-scoring compounds as viable binders supported by CmDock docking calculations considering the QuickProp QPlogS descriptor that indicates possible soluble compounds. Namely, solubility forms one of the serious problems when transitioning from in silico enrichment lists towards physical samples for in vitro evaluation. We supply full lists of hit compounds in the Supplementary Materials for the reader’s benefit, especially if alternative compound selection criteria are desired, all with the aim of providing the medicinal chemistry community with a viable prioritisation library of potential 3CL^pro^ inhibitors tailored for further molecular dynamics (MD) studies [61] as well as experimental design and development. Namely, reported enriched lists of compounds can serve as starting points, whereas compounds can be purchased commercially or be a subject of a synthetic campaign. Compounds can thus serve as experimental decoys or, if successful, progress as leads or probes.

## Figures and Tables

**Table 1:** Summary of dominant SARS-CoV-2 variants and relevant mutations.

Variant ^1^	Alternative Name	Sprot/ All Mutations	Key Mutations	Comment	3CL^pro^/PL^pro^ Mutations ^2^
B.1.1.7	UK Variant	8/23	E69/70 del 144Y del N501Y (RBD interface) A570D P681H	higher transmissibility	none/A1708D
B.1.351	South African Variant	9/21	K417N (RBD) E484K (RBD) N501Y (RBD) orf1b del	escape host immune response	none/K1655N
P.1	Brasil Variant	10/17	K417N/T (RBD) E484K (RBD) N501Y (RBD) orf1b del	under research	none/K1795Q
B.1.617	Indian Variant	7/23	G142D E154K L452R (RBD) E484Q (RBD) D614G P681R Q1071H	under research	none/under research

^1^ Other known variants are COH.20G, S Q677H (Midwest variant) and L452R, B1429; ^2^ The mutations on PL^pro^ are located far outside the enzyme’s active site.

**Table 2:** Identified top-scoring compounds in the HTVS on the SARS-CoV-2 main protease for further compound prioritisation in biological evaluation experiments.

no	Structure	Mr (g/mol)	Cluster/QPlogS ^1^	CmDock Docking Score ^2^	Classification ^3^
1	* 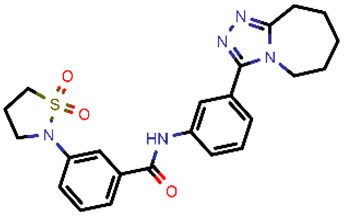 *	451.54	5/−6.42	−32.51	*general*
2	* 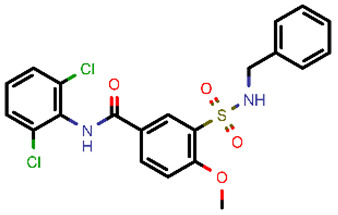 *	465.35	5/−6.22	−29.02	*general*
3	* 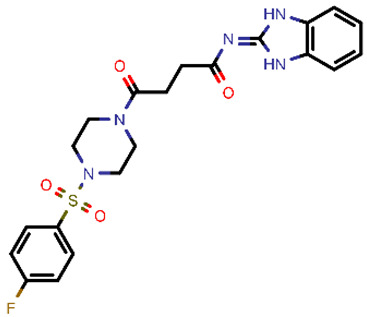 *	459.49	5/−3.89	−26.80	*viral_cys_prot*
4	* 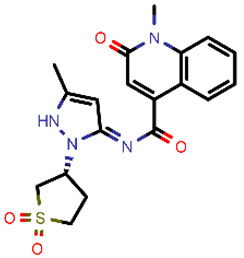 *	400.45	4/−4.54	−25.58	*viral_cys_prot*
5	* 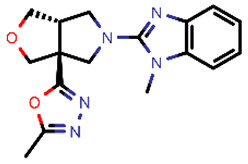 *	325.37	5/−3.59	−25.53	*viral_cys_prot*
6	* 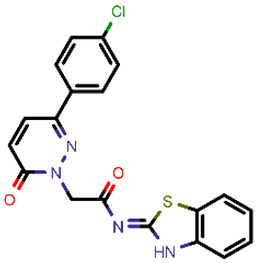 *	396.85	5/−6.47	−25.05	*general*
7	* 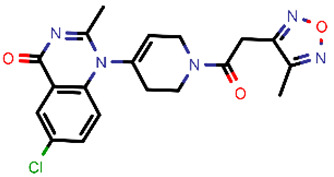 *	399.83	2/−3.44	−24.76	*general*
8	* 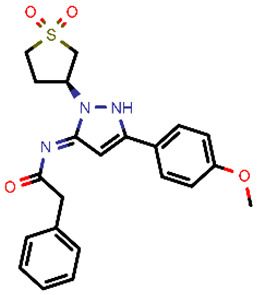 *	425.50	4/−5.38	−24.51	*general*
9	* 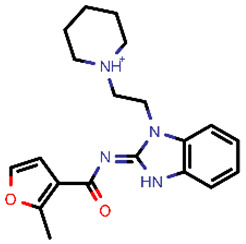 *	353.44	5/−4.40	−24.17	*general*
10	* 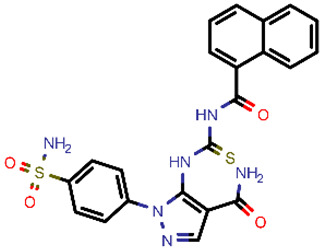 *	494.55	5/−5.60	−24.01	*general*
11	* 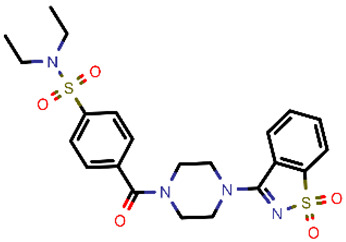 *	490.60	5/−2.87	−23.98	*general*
12	* 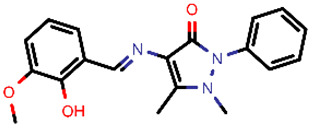 *	337.37	5/−4.75	−23.61	*viral_cys_prot*
13	* 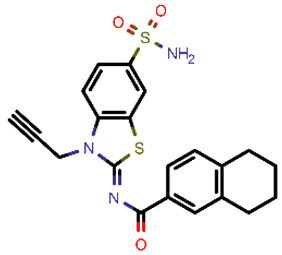 *	425.52	5/−5.66	−23.53	*general*
14	* 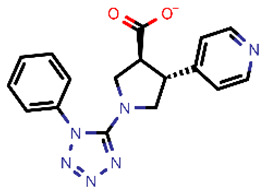 *	335.34	5/−3.90	−23.26	*general*
15	* 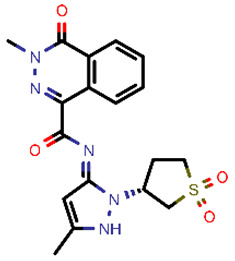 *	401.44	4/−5.00	−23.18	*general*

^1^ QPlogS Predicted aqueous solubility, log S. S in mol dm^−3^ is the solute concentration in a saturated solution in equilibrium with the crystalline solid (recommended value range by QuickProp is between –6.5 and –0.5); ^3^ CmDock INTER-molecular docking score.; ^3^ As per NeuralNet model.

**Table 3:** Machine learning model accuracy comparison table.

Macro F1/mse ^1^	NeuralNet ^2^	XGB ^2^	Linear ^2^	Majority/Average ^3^
Classification	0.895 ± 0.05	0.889 ± 0.014	0.667 ± 0.015	0.283 ± 0.001
Regression	0.002 ± 0.001	0.003 ±0.001	0.012 ± 0.002	0.005 ± 0.001

^1^ macro F1 for classification and mse for regression; ^2^ Learner; ^3^ majority class classifier in classification and average of the training target space in a regression model.

## Data Availability

Data is contained within the article or supplementary materials.

## Supplementary Materials

Details on target preparation and virtual screening. References [62] and [63] are cited in the supplementary materials.

## Sample Availability

Not available (compounds are commercially available).

## Abbreviations

3CL^pro^	3C-like protease
MD	Molecular Dynamics
VS	Virtual Screening
HTVS	High-Throughput Virtual Screening
LIE	Linear Interaction Energy
Xgb	Gradient boosting framework library
