# Supplementary material for: Prioritisation of Compounds for 3CLpro Inhibitor Development on SARS-CoV-2 Variants
Source: Molecules. 2021 May 18;26(10):3003. doi: 10.3390/molecules26103003 (PMC8158358; doi:10.3390/molecules26103003)
Supplement: Supplementary file 1 [file molecules-26-03003-s001.zip › COVID_SI_3clpro_compound_prioritisation_classification_jukic_et_al_Supplementary Materials.pdf]

molecules

MDPI

Supporting Information

Prioritisation of Compounds for 3CL^{pro} Inhibitor Development on SARS-CoV-2 Variants

Marko Jukič [1,2], Blaž Škrlj [3], Gašper Tomšič [4], Sebastian Pleško [5], Črtomir Podlipnik [6]* and Urban Bren [1,2]*

[1] Laboratory of Physical Chemistry and Chemical Thermodynamics, Faculty of Chemistry and Chemical Engineering, University of Maribor, , Smetanova ulica 17, SI-2000 Maribor, Slovenia; marko.jukic@um.si
[2] Faculty of Mathematics, Natural Sciences and Information Technologies, University of Primorska, Glagoljaška 8, SI-6000 Koper, Slovenia
[3] Institute Jožef Stefan, Jamova cesta 39, SI-1000 Ljubljana, Slovenia; blaz.skrlj@ijs.si
[4] Independent Researcher, Cesta Cirila Kosmača 66, SI-1000 Ljubljana, Slovenia; gasper.tomsic@icloud.com
[5] Erudio, Litostrojska cesta 40, SI-1000 Ljubljana, Slovenia; sebastian.plesko@erudio.si
[6] Faculty of Chemistry and Chemical Technology, University of Ljubljana, Večna pot 113, SI-1000 Ljubljana, Slovenia

* Correspondence: crtomir.podlipnik@fkkt.uni-lj.si (Č.P.); urban.bren@um.si (U.B.); Tel: +386-41-440-198 (Č.P.); Tel: +386-2-22-94-421 (U.B.)

1. Target preparation

DOCKING SITE

Total volume 3106.25 A^3

Cavity #1 Size=24850 points; Vol=3106.25 A^3; Min=(-33.5,-53.5,-8.5); Max=(-13,-26.5,12); Center=(-24.4138,-38.9632,-0.179235); Extent=(20.5,27,20.5) [62]

2. ROC study

We calculated the receiver operating characteristic (ROC) curve to validate the performance of classifier docking method. A set of known SARS-CoV 3CL^{pro} inhibitors from ChEMBL database (https://www.ebi.ac.uk/chembl/g/#browse/activities/filter/target_chembl_id%3ACHEMBL3927%20AND%20standard_type%3A(%22IC50%22; < 100 μM) with experimental values was concatenated with decoy compounds that were calculated using DUD-E: A Database of Useful (Docking) Decoys. Upon using 1% (2900 molecules in total) and 10 % (290 molecules in total) of actives in the test database, we obtained a ROC AUC of 0.80 and 0.79, respectively.

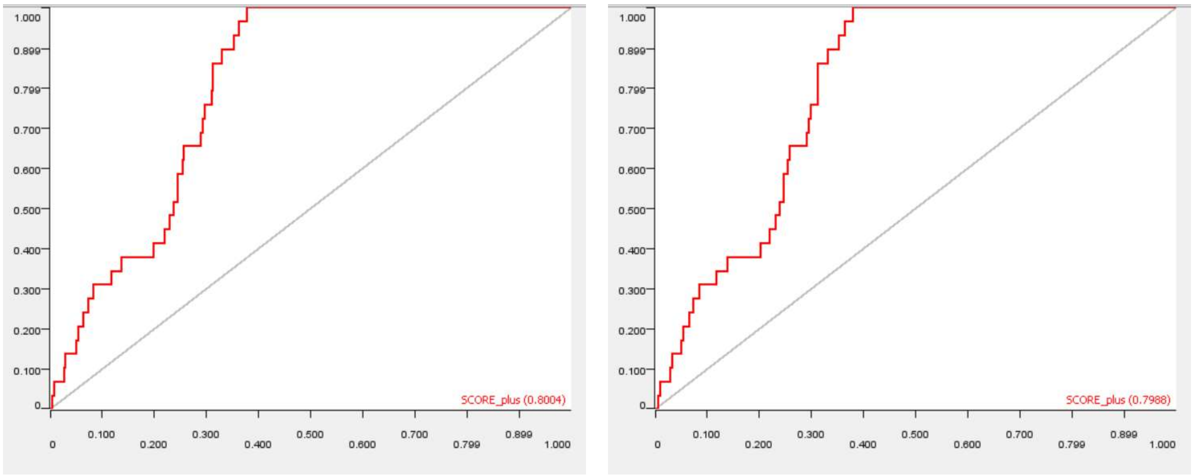

Figure S1: ROC curves for 1% (left) and 10% (right) actives in the complete set.

Furthermore, we employed the activity data from PostEra Covid Moonshot project (https://covid.postera.ai/covid/activity_data, accessed on 8.5.2021). We selected compounds with pIC_{50} above 7 as true actives and compounds with pIC_{50} up to 4.00436 as inactives or experimental decoys (compounds with no data were left out). When using 2% (due to

availability of experimental decoys; 446) and 10% of actives in the test set, we obtained a ROC AUC of 0.605 and 0.6115, respectively, indicating the docking protocol can identify active compounds and produce enriched libraries.

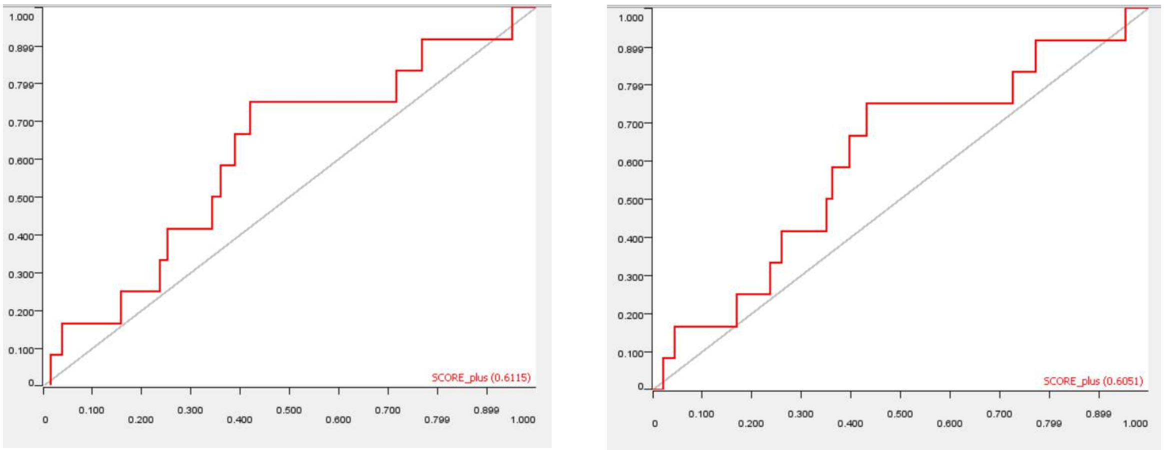

Figure S2: ROC curves for 2% (left) and 10% (right) actives in the complete test set (PostEra COVID Moonshot project).

Postera true actives (https://covid.postera.ai/covid/activity_data):

STRUCTURE	***NAME (PostEra)*, SMILES**
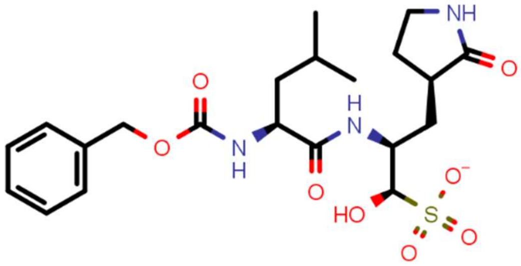	MSK-46727e7b-1<br><br>CC(C)C[C@H](NC(=O)OCc1ccccc1)C(=O)N[C@@H](C[C@H]1CCNC1=O)[C@H](O)S(=O)(=O)[O-]
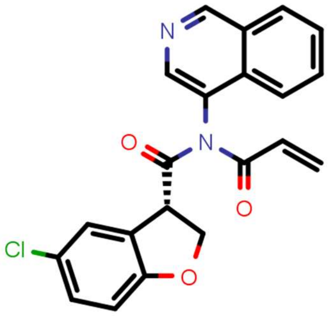	MAT-POS-e69ad64a-2<br><br>C=CC(=O)N(C(=O)[C@@H]1COc2ccc(Cl)cc21)c1cncc2ccccc12

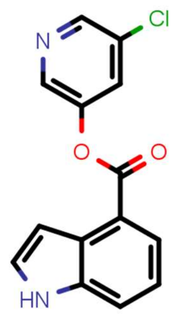	ALP-POS-c59291d4-5<br><br>O=C(Oc1cncc(Cl)c1)c1cccc2[nH]ccc12
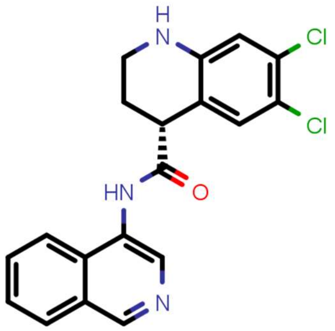	MAT-POS-f9802937-8<br><br>O=C(Nc1cncc2ccccc12)[C@@H]1CCNc2cc(Cl)c(Cl)cc21
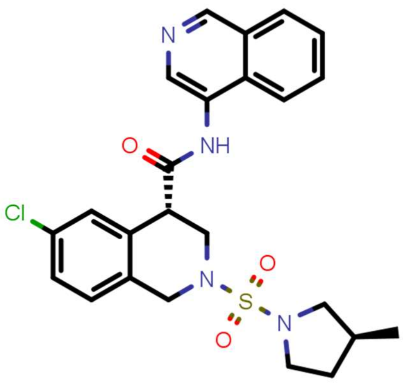	MAT-POS-4223bc15-11<br><br>C[C@H]1CCN(S(=O)(=O)N2Cc3ccc(Cl)cc3[C@H](C(=O)Nc3cncc4ccccc34)C2)C1
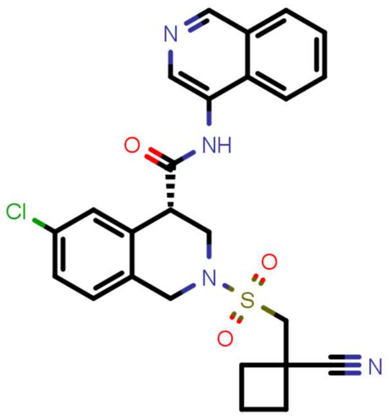	MAT-POS-4223bc15-12<br><br>N#CC1(CS(=O)(=O)N2Cc3ccc(Cl)cc3[C@H](C(=O)Nc3cncc4ccccc34)C2)CCC1

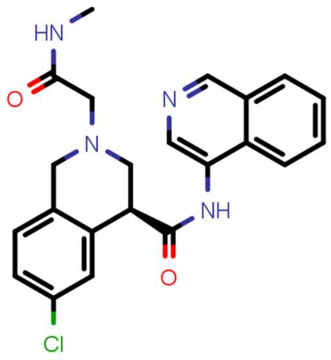	MAT-POS-4223bc15-23<br><br>CNC(=O)CN1Cc2ccc(Cl)cc2[C@H](C(=O)Nc2cncc3ccccc23)C1
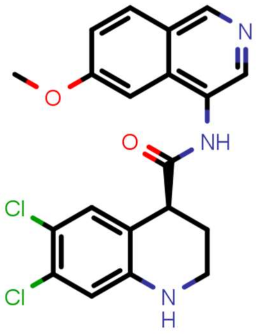	MAT-POS-ec6d90b7-6<br><br>COc1ccc2cncc(NC(=O)[C@H]3CCNc4cc(Cl)c(Cl)cc43)c2c1
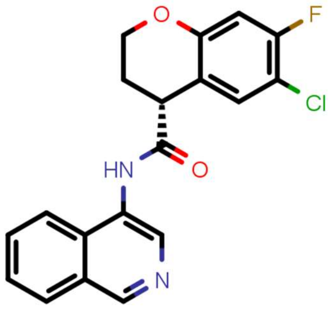	MAT-POS-a13804f0-3<br><br>O=C(Nc1cncc2ccccc12)[C@@H]1CCOc2cc(F)c(Cl)cc21

3. HTVS results

Supplied smiles, all other data is in the supplied xls file.

no	smiles	score
1	O=C(c1ccc2c(c1)C(=O)N(C2=O)c1c(C)n(n(c1=O)c1ccccc1)C)c1ccc2c(c1)C(=O)N(C2=O)c1c(C)n(n(c1=O)c1ccccc1)C	-22,8019
2	Clc1ccc(cc1S(=O)(=O)Nc1c(C)n(n(c1=O)c1ccccc1)C)C(=O)Nc1ccc(cc1)C(C)(C)C	-21,9941
3	O=C(c1ccc(c(c1)S(=O)(=O)Nc1c(=O)n(n(c1C)C)c1ccccc1)Cl)Nc1ccc2c(c1)cccc2	-21,0602
4	COc1ccc(cc1S(=O)(=O)Nc1c(C)n(n(c1=O)c1ccccc1)C)c1nnnn1CC	-20,8952
5	Cn1c(nnc1[C@@H]1C[C@@H](C1)[NH3+])Cn1c(nc2c1cccc2)N1CCCC1	-20,5798

6	Cc1cn(c(=O)nc1N1CCCCC1)c1nc(oc1c1ccccc1)c1ccccc1	-21,0488
7	Cn1c(=O)n(C)c(=O)c2c1nc(n2CCSc1nc2c(s1)cccc2)N1CCN(CC1)c1ccccc1	-25,6926
8	Cn1c(nnc1[C@@H]1C[C@H](C1)[NH3+])Cn1c(nc2c1cccc2)N1CCCC1	-22,5395
9	Nc1ccccc1c1scc(n1)c1cc(n(c1C)c1ncn[nH]1)C	-22,4204
10	[S-]c1nnc(n1c1cnn(c1)Cc1ccccc1)C1CCN(CC1)S(=O)(=O)c1ccccc1	-25,6427
11	COc1cc(ccc1O)c1c/c(=N/[C@H](C(=O)[O-])Cc2nc[nH]c2)/c2c(o1)cc(c(c2)Cl)C	-22,0924
12	Clc1cccc(c1)N1CCN(CC1)c1nc2c(n1CCSc1nc3c([nH]1)cccc3)c(=O)[nH]c(=O)n2C	-21,0076
13	Clc1cccc(c1)N1CCN(CC1)c1nc2c(n1CCSc1nc3c(o1)cccc3)c(=O)[nH]c(=O)n2C	-23,3288
14	CCOC(=O)c1ccccc1NC(=O)N[C@H](c1c(sc2c1CCCC2)n1cccc1)C	-24,0728
15	Clc1cccc(c1)N1CCN(CC1)c1nc2c(n1CCSc1nnnn1c1ccccc1)c(=O)[nH]c(=O)n2C	-20,8048
16	[S-]c1nnc(n1c1cnn(c1)Cc1ccccc1)[C@H]1CCCN(C1)S(=O)(=O)c1ccccc1	-22,9086
17	Clc1ccc(c(c1)Cl)Cn1ncc(c1)n1c([S-])nnc1C1CCN(CC1)S(=O)(=O)C	-24,2461
18	Cc1nn(c(c1N1CCN(CC1)C(=O)c1[nH]nc(c1)c1ccccc1)C)c1ccccc1	-22,6734
19	CCN(S(=O)(=O)c1ccc(cc1)C(=O)N1CCN(CC1)C1=NS(=O)(=O)c2c1cccc2)CC	-23,9761
20	[S-]c1nnc(n1c1cnn(c1)Cc1ccccc1)[C@H](n1nccc1C)C	-23,2749
21	Cc1nn(c(c1N1CCN(CC1)C(=O)c1[nH]nc(c1)c1cccs1)C)[C@H]1CCS(=O)(=O)C1	-23,0223
22	CC[C@H](c1c(sc2c1CC[N@@H+](C2)CC)n1cccc1)NC(=O)Nc1ccc(c(c1)C)C	-21,6719
23	Cc1cnn(c1)[C@@H](c1nnc(n1c1cnn(c1)Cc1ccccc1)[S-])C	-21,3904
24	Clc1ccccc1Cn1c(nc2c1c(=O)n(C)c(=O)n2C)N1CC[NH2+]CC1	-20,5788
25	c1ccc(cc1)c1[nH+]c(cn1c1sc(nc1c1ccccc1)c1ccccc1)c1ccccc1	-23,483
26	Clc1ccc(cc1)Cn1ncc(c1)n1c([S-])nnc1[C@@H]1CCCN(C1)S(=O)(=O)C	-21,0243
27	COCc1cc(C)nc2c1c(n1cccc1)c(s2)c1ccc(cc1)C#N	-20,5256

28	[O-]C(=O)[C@@H]1CN(C[C@H]1c1ccncc1)c1nnnn1c1ccccc1	-23,2589
29	O=C(c1c(C)nn(c1n1cccn1)C)NC[C@H]1[NH2+]CCc2c1cccc2	-20,7071
30	O=C(c1cnn(c1n1cccc1)c1ccccc1F)Nc1ccc2c(c1)OCO2	-22,7862
31	O=C(Nc1cc(C)cc(c1)C)CSc1nnc(n1Cc1ccccc1)n1cccc1	-20,8099
32	Cc1cnn(c1)[C@H](c1nnc(n1c1cnn(c1)Cc1ccccc1)[S-])C	-21,0524
33	Clc1ccc2c(c1)c(=O)nc(n2C1=CCN(CC1)C(=O)Cc1nonc1C)C	-24,7638
34	O=C(c1noc(c1)c1ccco1)N1CCN(CC1)c1c(C)nn(c1C)c1ccccc1	-20,8548
35	O=C(N[C@H](c1c(sc2c1CCCC2)n1cccc1)C)Nc1cccc(c1)Br	-21,6433
36	Cc1ccc(cc1)c1[nH+]c(cn1c1sc(nc1c1ccccc1)c1ccccc1)c1ccccc1	-22,0427
37	CCc1nc(=O)c2c(n1C1=CCN(CC1)C(=O)C[C@H]1C(=O)NCC[N@H+]1C)cccc2	-22,0768
38	C[C@@H]1CCCN(C1)C(=O)[C@@H]1CCN(CC1)C1=NS(=O)(=O)C(=C1C)c1ccc(c(c1)C)C	-21,0378
39	CCS(=O)(=O)c1sc(c(n1)S(=O)(=O)c1ccc(cc1)Cl)N1CCCCCC1	-21,1373
40	Clc1ccc(cc1)S(=O)(=O)NCc1c(n2cccc2)n(c(c1C)C)Cc1ccccc1	-20,8832
41	Cc1nnc(o1)[C@]12COC[C@@H]2CN(C1)c1nc2c(n1C)cccc2	-25,5332
42	O=C(N1CCN(CC1)c1c(C)nn(c1C)[C@@H]1CCS(=O)(=O)C1)CCc1c(C)noc1C	-21,8208
43	CC(=O)Nc1ccc(cc1)NC(=O)c1cnn(c1n1cccc1)c1ccccc1	-20,9442
44	O=C(Cn1c(=O)n(c2c1cccc2)C1=CCCCC1)NCc1ccc(cc1)NC(=O)C	-20,9659
45	O=C(Nc1ccc2c(c1)OCO2)CSc1nnc(n1c1ccc(cc1)C)N1CCCC1	-21,0771
46	NC(=O)C1CCN(CC1)C(=O)Cn1nc(C)c2c(c1=O)c(n1cccc1)n(n2)c1ccc(cc1)Cl	-21,5932
47	COc1cc(C)c(c(c1)C)S(=O)(=O)Nc1c(C)n(n(c1=O)c1ccccc1)C	-20,5389
48	S=C(NC(=O)c1cccc2c1cccc2)Nc1c(cnn1c1ccc(cc1)S(=O)(=O)N)C(=O)N	-24,0096
49	O=C(N=c1[nH]c2c([nH]1)cccc2)NC[C@@H]1CCC[C@H](C1)CNC(=O)N=c1[nH]c2c([nH]1)cccc2	-21,6642

50	O=C(c1c(/N=C/c2c3ccccc3cc3c2cccc3)sc2c1CCCC2)NCc1ccccc1	-23,3325
51	O=C(c1ccc(cc1)S(=O)(=O)N1CCOCC1)N1CCN(CC1)C1=NS(=O)(=O)c2c1cccc2	-20,6105
52	CCOC(=O)N1CCC(CC1)NC(=O)c1nnn(c1n1cccc1)Cc1ccccc1	-22,4857
53	O=C(c1cccc(c1)S(=O)(=O)Nc1c(C)n(n(c1=O)c1ccccc1)C)Nc1ccccc1C	-21,2497
54	c1ccc(nc1)c1noc(n1)[C@@H]1CCCN1c1nnnn1c1ccccc1	-22,8455
55	O=C([C@H]1CCCN(C1)c1nnc(s1)n1c(C)ccc1C)NCc1ccccc1Cl	-23,2297
56	O=C(c1nnn(c1n1cccc1)c1ccccc1)Nc1cnccc1C	-21,0582
57	O[C@H]1CCN(C1)c1nc2c(n1C)ccc(c2)C(=O)NCCc1ccc(o1)C	-22,4271
58	S=C1N[C@@H]([C@@H](N1c1ccc(cc1)OC1CCCC1)c1cc(n(c1C)c1nccs1)C)c1ccccn1	-22,2097
59	Cc1ccc(cc1)n1c(c2ccccc2)c(cc1c1ccccc1)/C=N/c1sc2c(c1C(=O)NC1CCCCC1)CCCC2	-22,7301
60	[NH3+]C[C@@H]1O[C@H](O[C@@H]2[C@H]([NH3+])C[C@@H]([C@@H]([C@@H]2O)O[C@H]2O[C@@H](CO)[C@H]([C@H]([C@H]2O)[NH3+])O)[NH3+])[C@@H]([C@@H]([C@@H]1O)O)O	-29,0171
61	COc1ccc(cc1)c1nc(sc1C)n1c(C)c2c(c1C)c(C)nnc2C	-23,2024
62	Cc1cccc(c1)N1C(=S)N[C@@H]([C@@H]1c1cc(n(c1C)c1nccs1)C)c1ccccn1	-23,9945
63	S=C(Nc1c(C)nn(c1C)Cc1ccccc1C)N[C@@H]1C[C@H]2CC[C@@H](C1)[N@@H+]2C	-26,6451
64	OCCNc1nc(=O)n(cc1C)c1nc(sc1c1ccccc1)c1ccccc1	-20,5596
65	O=C(Nc1ccc2c(c1)OCO2)CSc1nnc(n1Cc1ccccc1)N1CCOCC1	-23,1729
66	Cc1sc(nc1c1ccc(cc1)Oc1ccccc1)n1c(C)c2c(c1C)c(C)nnc2C	-29,3019
67	CCOc1ccc(cc1)S(=O)(=O)N(c1c(C)n(n(c1=O)c1ccccc1)C)Cc1ccc(cc1)C	-22,9799
68	O=C(C1CCN(CC1)c1nnc(s1)n1c(C)ccc1C)NCc1cccc(c1)Cl	-22,5213
69	Clc1ccc(cc1)N1C(=S)N[C@@H]([C@@H]1c1cc(n(c1C)c1nccs1)C)c1ccccn1	-23,5559
70	Clc1ccc(cc1)n1nc(/c(=N/C(=O)c2nnn(c2c2ccccc2)c2ccccc2)/s1)C(=O)N1CCOCC1	-30,9678
71	COc1cc(ccc1O)c1nc2n(c1Nc1cccc(c1)Cl)c(C)ccc2	-22,5591

72	S=C1N[C@H]([C@@H](N1c1ccc(c(c1)C)C)c1cc(n(c1C)c1nccs1)C)c1ccccn1	-22,9884
73	COc1cc(ccc1NS(=O)(=O)C)N1C(=S)N[C@H]([C@@H]1c1cc(n(c1C)c1nccs1)C)c1ccccn1	-21,5062
74	COc1ccc(cc1)c1[nH]n(/c(=N/C(=O)Cc2ccccc2)/c1)[C@H]1CCS(=O)(=O)C1	-24,5127
75	Cc1ccc(cc1)N1C(=S)N[C@@H]([C@@H]1c1cc(n(c1C)c1nccs1)C)c1ccccn1	-23,0373
76	COc1ccccc1Nc1c(nc2n1c(C)ccc2)c1ccc(cc1)O	-21,0136
77	Fc1ccc(cc1)[C@H]1C(=C(C(=O)N1c1c(C)n(n(c1=O)c1ccccc1)C)O)C(=O)c1ccc(cc1)C	-23,0111
78	S=C1N[C@H]([C@@H](N1c1ccc(c(c1)C)Br)c1cc(n(c1C)c1nccs1)C)c1ccccn1	-22,4476
79	COc1cc(ccc1OC)C(=O)/N=c/1\cc([nH]n1[C@H]1CCS(=O)(=O)C1)c1ccccc1	-20,8044
80	N#Cc1c(/N=C/c2c(OCc3ccc(cc3)Cl)ccc3c2cccc3)sc2c1CCCC2	-22,6305
81	Cc1ccc(cc1)N1C(=S)N[C@H]([C@@H]1c1cc(n(c1C)c1nccs1)C)c1ccccn1	-22,2832
82	Oc1ccccc1N1C(=S)N[C@H]([C@H]1c1cc(n(c1C)c1nccs1)C)c1ccccn1	-22,4993
83	S=C1N[C@H]([C@@H](N1c1ccccc1)c1cc(n(c1C)c1nccs1)C)c1ccccn1	-22,1985
84	S=C1N[C@H]([C@@H](N1c1ccc(c(c1)C)F)c1cc(n(c1C)c1nccs1)C)c1ccccn1	-22,1438
85	S=C1N[C@H]([C@@H](N1C1CCCC1)c1cc(n(c1C)c1nccs1)C)c1ccccn1	-23,0523
86	S=C(Nc1c(cnn1c1ccc(cc1C)C)C(=O)N)NC(=O)c1ccc(c(c1)S(=O)(=O)N1CCOCC1)Cl	-21,6727
87	COc1ccc(cc1)N1C(=S)N[C@H]([C@@H]1c1cc(n(c1C)c1nccs1)C)c1ccccn1	-20,6732
88	Fc1ccc(cc1)N1C(=S)N[C@@H]([C@H]1c1cc(n(c1C)c1nccs1)C)c1ccccn1	-23,6303
89	S=C1N[C@@H]([C@@H](N1c1ccc(cc1)Oc1ccccc1)c1cc(n(c1C)c1nccs1)C)c1ccccn1	-23,7284
90	Cc1sc(nc1c1ccc(c(c1)C)C)n1c(C)c2c(c1C)c(C)nnc2C	-22,538
91	Cc1cccc(c1)c1nn(cc1C(=O)N(c1nc2c(s1)cccc2)C[C@H]1CCCO1)c1ccccc1	-20,9917
92	COc1cc(ccc1OC)c1c/c(=N\c2sc(c(n2)c2cccs2)C)/c2c(o1)cccc2	-23,8736
93	N#Cc1c(/N=C/c2cn(c3c2cccc3)Cc2ccc3c(c2)cccc3)sc2c1CC[N@@H+](C2)C	-23,9072

94	Cc1ccnc(n1)Sc1oc(cc1Br)/C=N/c1sc2c(c1C(=O)Nc1ccccc1)CCCC2	-21,4688
95	O=C(Nc1cc(C)ccc1C)CCN1C(=S)N[C@H]([C@@H]1c1cc(n(c1C)c1nccs1)C)c1ccccn1	-25,8391
96	Oc1ccccc1N1C(=S)N[C@@H]([C@H]1c1cc(n(c1C)c1nccs1)C)c1ccccn1	-23,9707
97	COCC[NH+]1CCN(CC1)C(=O)c1cccc(c1)c1nn(c(n1)N(C)C)C	-20,7663
98	COc1ccc(cc1)c1cc(nn1[C@H]1CCS(=O)(=O)C1)C(=O)N=c1[nH]cc[nH]1	-20,9064
99	O=C1N[C@@H](C(=O)N1CC(=O)c1cc(n(c1C)c1nccs1)C)Cc1c[nH]c2c1cccc2	-25,3222
100	S=C1N[C@H]([C@@H](N1c1ccc2c(c1)OCO2)c1cc(n(c1C)c1nccs1)C)c1ccccn1	-21,0808
101	Brc1ccc(cc1)S(=O)(=O)Nc1nc2ccccc2nc1n1ccc(=NC(=O)c2ccccc2)cc1	-22,1721
102	Clc1ccc(cc1)N1C(=S)N[C@H]([C@H]1c1cc(n(c1C)c1nccs1)C)c1ccccn1	-20,923
103	Oc1ccccc1N1C(=S)N[C@@H]([C@@H]1c1cc(n(c1C)c1nccs1)C)c1ccccn1	-21,8087
104	Clc1ccc(cc1)NC(=O)c1c(/N=C/c2ccc3c(c2)Sc2c(N3C)cccc2)sc2c1CC[C@H](C2)C(C)(C)C	-25,7525
105	COc1cccc(c1)N(C(=O)c1ccc(cc1)NC(=O)C)c1nc2c(s1)cccc2	-21,3127
106	O=C(Nc1ccc(cc1)C)CCN1C(=S)N[C@H]([C@@H]1c1cc(n(c1C)c1nccs1)C)c1ccccn1	-24,6782
107	COc1cccc(c1O)/C=N/c1c(C)n(n(c1=O)c1ccccc1)C	-23,6146
108	Clc1ccc(c(c1)Cl)COc1ccccc1/C=N/c1sc2c(c1C(=O)NCc1ccccc1)CCCC2	-21,944
109	Oc1ccccc1/C=N/C1=CC=C(CC1)Cc1ccc(cc1)/N=C/c1ccccc1O	-22,0927
110	Clc1ccc2c(c1)oc(=O)n2CC(=O)c1cc(n(c1C)c1nccs1)C	-21,7132
111	O=C(N=c1[nH]c2c([nH]1)cccc2)CSCc1nc2sc(cc2c(=O)[nH]1)C	-22,956
112	C[NH2+][C@@H]1[C@@H](O[C@H]2[C@H]([C@H]1O)O[C@H]([C@H](C2)[NH3+])O[C@@H]1[C@H]([NH3+])C[C@@H]([C@@H]([C@@H]1O)O)[NH3+])O[C@@H]1O[C@H](CO)[C@@H]([C@@H]([C@@H]1O)O)[NH3+]	-31,2203
113	O=C(N1CCN(CC1)S(=O)(=O)c1ccc(cc1)F)CCC(=O)N=c1[nH]c2c([nH]1)cccc2	-26,7982
114	N#Cc1c(/N=C/c2ccc(s2)c2cccs2)sc2c1CC[C@H](C2)C	-21,3807
115	S=C1N[C@H]([C@@H](N1c1ccc(cc1)Oc1ccccc1)c1cc(n(c1C)c1nccs1)C)c1ccccn1	-21,4658

116	N#Cc1c(/N=C/c2ccc(s2)c2cccs2)sc2c1CC[C@@H](C2)C	-21,2289
117	CC(=O)N[C@H](C(=O)NC1CCN(CC1)c1nnnn1c1ccccc1)Cc1ccccc1	-22,0681
118	O=C(c1snnc1C)/N=c/1\cc([nH]n1[C@H]1CCS(=O)(=O)C1)c1ccccc1	-22,1781
119	Clc1ccc(cc1)NC(=O)c1c(/N=C/c2ccc(o2)N2CCOCC2)sc2c1CC[C@H](C2)C(C)(C)C	-22,9963
120	N#Cc1ccccc1COc1ccc(cc1/C=N/c1sc2c(c1C(=O)Nc1ccc(cc1)Cl)CC[C@H](C2)C(C)(C)C)Br	-21,132
121	C[C@H]1C[C@H](C)CN(C1)S(=O)(=O)c1cccc(c1)C(=O)N=C(c1ccc(cc1)N(C)C)c1ccc(cc1)N(C)C	-24,1544
122	O=C(Nc1ccc(cc1)C)CCN1C(=S)N[C@@H]([C@H]1c1cc(n(c1C)c1nccs1)C)c1ccccn1	-22,8567
123	N#Cc1c(/N=C/c2c(OCc3ccc(cc3)Br)ccc3c2cccc3)sc2c1CC[C@H](C2)C(C)(C)C	-20,5024
124	COc1ccc(cc1)c1c/c(=N\c2sc(c(n2)c2ccccc2)C)/c2c(o1)cccc2	-23,0855
125	C[NH2+][C@H]([C@@H]1CC[C@@H]([C@@H](O1)O[C@@H]1[C@H]([NH3+])C[C@@H]([C@@H]([C@@H]1O)O[C@H]1OC[C@]([C@H]([C@H]1O)[NH2+]C)(C)O)[NH3+])[NH3+])C	-21,149
126	Clc1ccc(cc1)S(=O)(=O)c1ccc(o1)C(=O)/N=c\1/s[nH]c(n1)c1ccccc1	-21,4012
127	O=C(Nc1ccc(cc1)C)CCN1C(=S)N[C@H]([C@H]1c1cc(n(c1C)c1nccs1)C)c1ccccn1	-21,3416
128	O=C(c1nn(C)c(=O)c2c1cccc2)/N=c/1\cc([nH]n1[C@@H]1CCS(=O)(=O)C1)C	-23,1844
129	COCC(=O)Nc1ccc(cc1Cl)N1C(=S)N[C@H]([C@@H]1c1cc(n(c1C)c1nccs1)C)c1ccccn1	-21,3439
130	O=C(c1cc(=O)n(c2c1cccc2)C)/N=c/1\cc([nH]n1[C@@H]1CCS(=O)(=O)C1)C	-25,5837
131	[NH3+]C[C@@H]1O[C@H](O[C@H]2[C@H]([NH3+])C[C@H]([C@H]([C@H]2O)O[C@H]2O[C@@H](CO)[C@H]([C@H]([C@H]2O)[NH3+])O)[NH3+])[C@@H](C[C@@H]1O)[NH3+]	-27,7312
132	O=C(c1sc(c(c1)c1ccccc1)N1CCOCC1)Nc1ccc(cc1)n1cnnn1	-21,7378
133	CCCS(=O)(=O)c1nnc(s1)/N=C(/COc1ccc(cc1)Br)\O	-20,6256
134	Clc1ccc(cc1)NC(=O)c1c(/N=C/c2c(OCc3ccc(cc3)Br)ccc3c2cccc3)sc2c1CC[C@H](C2)C(C)(C)C	-20,8778
135	O=C1N(c2ccc3c(c2)sc(n3)Sc2ccc(o2)/C=N/c2c(C)n(n(c2=O)c2ccccc2)C)C(=O)c2c1cccc2	-20,996
136	[NH3+]C[C@@H]1O[C@@H](O[C@@H]2[C@H]([NH3+])C[C@@H]([C@H]([C@H]2O)O[C@@H]2O[C@H](CO)[C@@H]([C@@H]([C@@H]2O)[NH3+])O)[NH3+])[C@@H]([C@H]([C@H]1O)O)O	-27,5638
137	O=C(c1cccc(c1)Nc1ncccn1)/N=c/1\cc([nH]n1[C@@H]1CCS(=O)(=O)C1)C	-22,3551

138	Fc1ccc2c(c1)cc(n2C)C(=O)/N=c/1\cc([nH]n1[C@H]1CCS(=O)(=O)C1)C	-21,8077
139	CCOc1ccc(cc1)N1C(=S)N[C@H]([C@H]1c1cc(n(c1C)c1nccs1)C)c1ccccn1	-20,7452
140	O1CCN(CC1)c1nnc(n1Cc1ccccc1)SCc1noc(c1)c1ccco1	-21,0275
141	CCOc1cc(/C=N/c2c(C)n(n(c2=O)c2ccccc2)C)ccc1O	-22,4696
142	O=C(c1c(/N=C/c2c(O)ccc3c2cccc3)sc2c1CCCC2)NCc1ccccc1	-20,7485
143	Oc1ccc(cc1)n1nnnc1SCC(=O)c1cc(n(c1C)c1nccs1)C	-21,2242
144	COCCn1ccc2c1cccc2C(=O)/N=c/1\cc([nH]n1[C@H]1CCS(=O)(=O)C1)C	-23,8236
145	S=C(Nc1nnc(c(=O)[nH]1)C)/N=C/c1ccc(cc1)N(C)C	-20,6842
146	N#Cc1ccccc1NC(=O)CSc1nc2ccccc2c(=O)n1c1[n-]cnn1	-20,5326
147	Brc1cc(oc1Sc1ccccc1)/C=N/c1sc2c(c1C(=O)NCc1ccco1)CCCC2	-20,6364
148	COc1cccc(c1)N(C(=O)c1ccncc1)c1nc2c(s1)cccc2	-20,8889
149	COc1cc(ccc1O)c1nc2n(c1/N=C/c1cn(c3c1cccc3)Cc1ccccc1Cl)cccc2	-25,691
150	O=C(c1cccc(c1)N1CCCS1(=O)=O)Nc1cccc(c1)c1nnc2n1CCCCC2	-32,513
151	C#CCn1/c(=N/C(=O)c2ccc3c(c2)CCCC3)/sc2c1ccc(c2)S(=O)(=O)N	-23,5295
152	Oc1cc(C)c(c(c1)O)/C=N/c1c(=O)n(n(c1C)C)c1ccccc1	-22,5703
153	COc1ccc2c(c1)s/c(=N\C(=O)CCCn1sc3c(c1=O)cccc3)/[nH]2	-22,7957
154	Clc1ccc(cc1)NC(=O)c1c(/N=C/c2cc(c(o2)Sc2ncccn2)Br)sc2c1CCCC2	-24,5014
155	Clc1cccc(n1)C(=O)/N=c/1\cc([nH]n1[C@H]1CCS(=O)(=O)C1)c1ccccc1	-21,0956
156	COC(=O)c1c(NC(=S)NCc2cn(nc2c2ccccc2)c2ccccc2)sc2c1CCC2	-22,2075
157	Cc1ccc(cc1)c1n[nH]cc1/C=N/c1sccc1C(=O)Nc1ccccc1C	-21,9494
158	O=C([C@H]1CCCN(C1)c1nnc(s1)n1c(C)ccc1C)NCCC[NH+]1CCCCCC1	-20,9396
159	O=C(Nc1ccccc1C)CCN1C(=S)N[C@H]([C@H]1c1cc(n(c1C)c1nccs1)C)c1ccccn1	-20,7708

160	Cc1ccc(cc1)N1[NH2+][C@](NC1=S)(C)c1ccccc1Br	-22,8459
161	COc1ccc(cc1S(=O)(=O)NCc1ccccc1)C(=O)Nc1c(Cl)cccc1Cl	-29,0161
162	O[C@H](C[NH+](C1CCCCC1)C1CCCCC1)Cn1c(c2ccccc2)c(c2c1c1ccccc1cc2)c1ccccc1	-24,7942
163	O=C(c1cc(nc2c1c(C)no2)C(C)C)N=c1[nH]c2c([nH]1)cccc2	-22,0301
164	O=C(c1c(/N=C/c2c[nH]c3c2cccc3)sc2c1CCCC2)Nc1ccccc1	-25,1821
165	OCCCN1C(=S)N[C@H]([C@@H]1c1cc(n(c1C)c1nccs1)C)c1ccccn1	-20,7201
166	Clc1ccc(cc1)c1noc(n1)CCCSc1nnc(n1Cc1ccccc1)N1CCOCC1	-20,5987
167	COc1cc(ccc1O)c1nc2n(c1/N=C/c1ccc(o1)c1cccc(c1C)Cl)cccc2	-22,0229
168	O=C(C1CCN(CC1)c1ccc(nn1)Cl)N=c1[nH]c2c([nH]1)cccc2	-21,7947
169	O=C(c1sc2c(c1N)cccn2)/N=c/1\sc2c([nH]1)cccc2	-21,5002
170	Clc1ccc(cc1)c1ccc(=O)n(n1)CC(=O)/N=c/1\sc2c([nH]1)cccc2	-25,0465
171	O=C(c1sc(c(c1N)c1sc2c(n1)cccc2)Nc1ccccc1)c1ccccc1	-21,3489
172	Clc1ccc(cc1)C(=O)c1sc(c(c1N)c1nc2c(s1)cccc2)Nc1cc(C)ccc1C	-21,8297
173	O/C(=N\c1scc(n1)C[N@H+]1CCc2c(C1)cccc2)/c1cc(=O)c2c(o1)cccc2	-21,4759
174	CCOC(=O)c1ccc(s1)NC(=O)c1cccc(c1)S(=O)(=O)N1CCCc2c1cccc2	-22,1124
175	O=C1Nc2nc(nn2[C@H](C1)c1ccc(cc1)Cl)/N=C(/COc1ccc2c(c1)cccc2)\O	-23,0783
176	Fc1ccc(cc1)NC(=S)Nc1sc2c(c1C(=O)N)CCCC2	-22,244
177	C[C@@H]1CCc2c(C1)sc(c2C(=O)N)NC(=S)NC(=O)c1sc2c(c1Cl)ccc(c2)Cl	-21,5284
178	COc1ccc(cc1)C(=O)/N=c/1\sn(nc1C(=O)Nc1ccccc1)c1ccc(cc1)Cl	-22,4954
179	CC(NC(=O)c1cccc(c1)Cn1/c(=N/C(=O)c2ccccc2F)/sc(c1C)C)C	-21,1394
180	Cc1cccc(c1)C(=O)NC(=S)Nc1sc2c(c1C(=O)N)CCCCC2	-23,0945
181	COc1ccc(cc1)c1[nH]n(/c(=N/C(=O)C(C)(C)C)/c1)[C@H]1CCS(=O)(=O)C1	-22,994

182	C[C@@H]1CCN(CC1)c1ccc(o1)/C=N/c1sc2c(c1C(=O)Nc1ccccc1)CCCC2	-21,9718
183	CCn1/c(=N/C(=O)c2ccc(cc2)S(=O)(=O)N2C[C@H](C)O[C@H](C2)C)/sc2c1ccc(c2)Cl	-21,7689
184	O=C(c1ccoc1C)/N=c/1\[nH]c2c(n1CC[NH+]1CCCCC1)cccc2	-24,1678
185	C1CC[C@H](C[NH2+]1)Cc1cnc/c(=N\c2ncc(s2)c2ccccc2)/[nH]1	-21,8052
186	Clc1cccc(c1)c1n[nH]/c(=N/C(=O)NCc2n(C)ncc2C2CC2)/s1	-21,2698
187	COC(=O)Nc1c(nc(n1c1sc(nc1c1ccccc1)c1ccccc1)c1ccccc1)c1ccccc1	-20,6814
188	Fc1ccc(cc1)c1c[nH]/c(=N\C(=O)c2[nH]nc3c2C[C@H](CC3)C(C)(C)C)/n1C	-20,6693
189	Cc1nn(c(c1)C)c1cccc(c1)C(=O)/N=c/1\cc([nH]n1[C@@H]1CCS(=O)(=O)C1)C	-22,0209
190	CC[N@@H+]1CCc2c(C1)sc(c2C(=O)N)NC(=S)NC(=O)c1c(Br)cnn1C	-20,5992
191	COc1ccc2c(c1)sc(n2)N1C(=O)c2c([C@H]1c1ccc(c(c1)OC)O)c(=O)c1c(o2)cccc1	-23,2908
192	Clc1ccc(cc1)COc1cccc(c1)/C=N/c1sc2c(c1C(=O)Nc1ccc(cc1)Cl)CC[C@@H](C2)C(C)(C)C	-21,1232
193	COc1cc(OC)ccc1C(=O)/N=c/1\sc2c(n1C)ccc(c2)S(=O)(=O)C	-20,8435
194	O=C(/N=c/1\cc([nH]n1[C@H]1CCS(=O)(=O)C1)C)CCc1c(C)n[nH]c1C	-20,5321
195	O1CCN(CC1)c1nnc(n1Cc1ccccc1)SCc1onc(n1)c1cscc1	-20,6485
196	Clc1ccc2c(c1)s/c(=N\C(=O)c1ccc(cc1)S(=O)(=O)N1CCCc3c1cccc3)/n2C	-23,7146
197	CC(=O)/N=C\1/CC(=CC(=C1)[NH3+])CCc1ccccc1	-20,6249
198	O=C(Cc1c(C)noc1C)N=c1[nH]c2c([nH]1)cccc2	-21,8791
199	[NH3+]C[C@H]1O[C@H](O[C@@H]2[C@@H]([NH3+])C[C@H]([C@H]([C@@H]2O)O[C@@H]2O[C@@H](CO)[C@H]([C@H]([C@@H]2O)[NH3+])O)[NH3+])[C@H](C[C@@H]1O)[NH3+]	-22,2492
200	[NH3+]C[C@@H]1O[C@H](O[C@@H]2[C@H]([NH3+])C[C@H]([C@@H]([C@@H]2O)O[C@H]2O[C@@H](CO)[C@H]([C@H]([C@H]2O)[NH3+])O)[NH3+])[C@@H]([C@@H]([C@@H]1O)O)O	-24,5993

4. DL Classification

Chompounds from ChEMBL with 3CL^{pro} activity data in csv format (SMILES and ChEMBL Standard value (IC_{50} in nM))

Cc1nc(S(=O)(=O)c2ccccc2)c(C#N)c(C)c1[N+](=O)[O-],13000
CCOC(=O)/C=C/[C@H](C[C@@H]1CCNC1=O)NC(=O)[C@H](CC=C(C)C)CC(=O)[C@@H](NC(=O)c1cc(C)on1)
C(C)C,70000
CSc1[nH]nc(NC(=O)c2cccs2)c1S(=O)(=O)c1ccccc1,15000
O=S(=O)(Cc1[nH]c(-c2ccc(Cl)s2)c[s+]1)c1cccs1.[Br-],18000
Cc1oc(C(C)(C)C)cc1-c1cc(NS(=O)(=O)c2cccs2)[nH]n1,10000
O=C(Oc1cncc(Cl)c1)c1ccco1,60
CCOC(=O)/C(C#N)=C/Nc1ccc(S(=O)(=O)c2ccc(/N=C/C(C#N)=C(\O)OCC)cc2)cc1,16000
CSc1nn(-c2c([N+](=O)[O-])c(C)nn2C)c(-c2cccs2)c1C#N,18000
O=[N+]([O-])c1ccc(S(=O)(=O)c2ccc(Cl)cc2)[n+]([O-])c1,15000
O=C(O)c1ccc(S(=O)(=O)c2cc(Br)c(O)c(Br)c2)cc1,16000
CCOC(=O)C(=CNc1ccc(S(=O)(=O)c2ccc(/N=C/C(C(=O)OCC)=C(\O)OCC)cc2)cc1)C(=O)OCC,32000
Cc1noc(NC(=O)c2ccc(-c3cc(C(F)(F)F)nn3C)s2)c1[N+](=O)[O-],5000
CC(Sc1nc(O)c(C#N)c(-c2ccccc2)n1)C(=O)Nc1ccc(Cl)cc1,300000
Cc1cc(O)nc(SCC(=O)Nc2ccc(Oc3ccc(Cl)cc3)cc2)n1,200000
CCCc1cc(O)nc(SCC(=O)Nc2ccc(Cl)cc2)n1,30000
O=C1C(=O)N(CC2COc3ccccc3O2)c2ccc(I)cc21,13500
O=C1C(=O)N(Cc2cc3ccccc3s2)c2c1cccc2[N+](=O)[O-],2000
Cc1nn(C)c(NCc2ccc(-c3cccs3)s2)c1[N+](=O)[O-],20000
CC(C)c1ccc(NC(=O)CSc2nccc(-c3csc(COc4ccccc4Cl)n3)n2)cc1,200000
Cc1nc(-c2nc(-c3ccnc(SCC(=O)Nc4ccc(Cl)cc4)n3)cs2)cs1,70794578438413.7
CSc1sc(-c2nc(C)cs2)c(C)c1-c1ccnc(SCC(=O)Nc2ccc(Cl)cc2)n1,11000
Cn1nc(-c2ccc(-c3ccnc(SCC(=O)Nc4ccc(Cl)cc4)n3)s2)cc1C(F)(F)F,100000000000000
O=C(CSc1nccc(-c2csc(-c3ccccc3)n2)n1)Nc1cc(Cl)cc(Cl)c1,3000
COC(OC)c1cc(O)nc(SCC(=O)Nc2ccc(C(F)(F)F)cc2)n1,4897788193684.46
COC(OC)c1cc(O)nc(SCC(=O)Nc2ccc(C(F)(F)F)cc2)n1,200000
Cc1cc(O)nc(SCC(=O)Nc2cc(Cl)ccc2Oc2ccccc2)n1,10000000000000
CSc1sc(-c2nc(C)cs2)c(C)c1-c1ccnc(SCC(=O)Nc2ccccc2Cl)n1,12000
Cc1cc(O)nc(SCC(=O)Nc2ccc(Oc3ccc(Cl)cc3)cc2)n1,4897788193684.46
Cc1noc(C)c1CN1C(=O)C(=O)c2cc(C#N)ccc21,7200
CC(C)(C)c1ccc(NC(=O)CSc2nc(O)c(C#N)c(C3CCCCC3)n2)cc1,2818382931264.45
O=C(CSc1nccc(-c2cc(-c3ccccc3)no2)n1)Nc1ccc(Cl)cc1,66069344800759.7
COc1cccc(-c2nc(SCC(=O)Nc3ccc([N+](=O)[O-])cc3)nc(O)c2C#N)c1,300000
O=C(CSc1nccc(-c2cc(-c3ccccc3Cl)no2)n1)Nc1ccc(Cl)cc1,66069344800759.7
CCCc1cc(O)nc(SCC(=O)Nc2ccc(Cl)cc2)n1,33113112148259.1
O=C(Oc1cncc(Cl)c1)c1cscn1,270
O=C(Oc1cncc(Cl)c1)c1ccc(-c2ccc(Cl)cc2)o1,63
O=C(Nc1ccc(Cl)cc1)c1ccc(CN2C(=O)C(=O)c3cc(I)ccc32)s1,12570
CCCCN1C(=O)C(=O)c2cc(C(N)=O)ccc21,19000
O=C1C(=O)N(Cc2cc3ccccc3s2)c2ccccc21,13110
O=C1C(=O)N(Cc2cc3ccccc3s2)c2cccc(Cl)c21,11200
CCCCN1C(=O)C(=O)c2cc(I)ccc21,66000
O=C(Oc1cncc(Br)c1)c1ccco1,50
O=C(Oc1cncc(Cl)c1)c1cc2ccccc2o1,170
C[C@H]1COC2=C1C(=O)C(=O)c1c2ccc2c1CCCC2(C)C,10100
Cc1coc2c1C(=O)C(=O)c1c-2ccc2c1CCCC2(C)CO,52000
O=C(Oc1cncc(Cl)c1)c1cc2ccccc2s1,95
C[C@H]1COC2=C1C(=O)C(=O)c1c2ccc2c1CCCC2(C)C,226700
COc1cccc(C(=O)Oc2cncc(Cl)c2)c1,340
COc1cccc(-c2nc(SCC(=O)Nc3ccc([N+](=O)[O-])cc3)nc(O)c2C#N)c1,3311311214825.91
COc1ccc(NC(=O)CSc2nc(O)cc(-c3ccccc3)n2)cc1OC,21877616239495.5
O=C(CSc1nccc(-c2cc(-c3ccccc3Cl)no2)n1)Nc1ccc(Cl)cc1,15000
Cc1nc(SCC(=O)Nc2ccc([N+](=O)[O-])cc2)nc(O)c1C,1000000000000
COc1cccc(-c2nc(SCC(=O)Nc3ccc(C(C)=O)cc3)nc(O)c2C#N)c1,16595869074375.6
O=C1C(=O)N(Cc2cc3ccccc3s2)c2c(Br)cccc21,980
O=[N+]([O-])c1ccc(S(=O)(=O)c2ccc([N+](=O)[O-])cc2)cc1,25000
Cc1cccc2c3c(ccc12)C1=C(C(=O)C3=O)[C@@H](C)CO1,14400
Cc1coc2c1C(=O)C(=O)c1c-2ccc2c1CCCC2(C)C,1600
CCC(Sc1nc(O)c(C#N)c(-c2cccc(OC)c2)n1)C(=O)Nc1ccc(C(C)=O)cc1,16595869074375.6
CSc1sc(-c2nc(C)cs2)c(C)c1-c1ccnc(SCC(=O)Nc2ccccc2Cl)n1,83176377110267.1
COc1ccc(-c2ccnc(SCC(=O)Nc3ccc(C(C)C)cc3)n2)cc1,2454708915685.03
CCOc1ccc(N2C(=O)CC(Sc3nc(C)cc(C)n3)C2=O)cc1,300000
O=C(CSc1nccc(-c2cc(-c3ccccc3)no2)n1)Nc1ccc(Cl)cc1,15000
Cc1nc(-c2nc(-c3ccnc(SCC(=O)Nc4ccc(Cl)cc4)n3)cs2)cs1,14000
CSc1sc(-c2nc(C)cs2)c(C)c1-c1ccnc(SCC(=O)Nc2ccc(Cl)cc2)n1,89125093813374.4
O=C1C(=O)N(Cc2ccc(C(=O)N3CCCCC3)s2)c2ccc(I)cc21,17500
O=C1C(=O)N(Cc2cc3ccccc3s2)c2ccc(I)cc21,950

O=C(Sc1nnc(C(F)(F)F)[nH]1)c1ccc(C#Cc2ccccc2)o1,3000
Cc1cc(S(=O)(=O)c2c([N+](=O)[O-])cc(C(F)(F)F)cc2[N+](=O)[O-])c(Cl)cc1Cl,300
CN1C(=O)C(=O)c2cc(C(N)=O)ccc21,71000
Cc1coc2c1C(=O)C(=O)c1c-2ccc2c1CCCC2(C)C,89100
Cc1cccc2c3c(ccc12)C1=C(C(=O)C3=O)[C@@H](C)CO1,1200
Nc1ncc(S(=O)(=O)c2ccc(Cl)cc2)c(N)n1,6000
Cc1cc(C(F)(F)F)nc(SCC(=O)Nc2ccc(Cl)cc2F)n1,500000
CCCCc1ccc(NC(=O)CSc2nc(O)c(C#N)c(C3CCCCC3)n2)c(C)c1,4897788193684.46
O=C(Cc1nccs1)c1nccs1,40000
Cc1coc2c1C(=O)C(=O)c1c-2ccc2c(C)cccc12,38700
C[C@H]1COC2=C1C(=O)C(=O)c1c2ccc2c1CCCC2(C)C,800
O=C(Oc1cncc(Cl)c1)c1cc2ccccc2[nH]1,65
C[C@H]1COC2=C1C(=O)C(=O)c1c2ccc2c1CCCC2(C)C,87600
CCOC(=O)/C=C/[C@H](C[C@@H]1CCNC1=O)NC(=O)[C@@H](CC(=O)[C@@H](NC(=O)c1cc(C)on1)C(C)C)Cc1ccccc1,45000
CC(C)C1=Cc2ccc3c(c2C(=O)C1=O)CCCC3(C)C,30000
O=C1C(=O)N(Cc2ccc(F)cc2Cl)c2ccc(I)cc21,9400
O=C1C(=O)N(Cc2cc3ccccc3s2)c2ccc(F)cc21,4820
O=[N+]([O-])c1cc(C(F)(F)F)ccc1S(=O)(=O)c1ccc(Cl)cc1,12000
Cc1ccc(S(=O)(=O)c2nc(C)c([N+](=O)[O-])c(C)c2C#N)cc1,13000
CCOC(=O)c1cnc(SCC(=O)Nc2ccc([N+](=O)[O-])cc2)nc1N,3981071705534.97
N#Cc1c(O)nc(SCC(=O)Nc2cccc(C(F)(F)F)c2)nc1-c1ccccc1,4897788193684.46
Cn1nc(-c2ccc(-c3ccnc(SCC(=O)Nc4ccc(Cl)cc4)n3)s2)cc1C(F)(F)F,10000
COc1cccc(-c2nc(SCC(=O)Nc3ccc(S(N)(=O)=O)cc3)nc(O)c2C#N)c1,24547089156850.3
COc1cccc(-c2nc(SCC(=O)Nc3ccc(C(C)=O)cc3)nc(O)c2C#N)c1,60000
CCOC(=O)c1cnc(SCC(=O)Nc2ccc([N+](=O)[O-])cc2)nc1N,250000
CCC(Sc1nc(O)c(C#N)c(-c2cccc(OC)c2)n1)C(=O)Nc1ccc(C(C)=O)cc1,60000
CC(C)c1ccc(NC(=O)CSc2nccc(-c3cccs3)n2)cc1,24547089156850.3
O=C(CSc1nccc(-c2cc(-c3ccc(Cl)cc3Cl)no2)n1)Nc1ccc(C(F)(F)F)cc1,66069344800759.7
O=C(CSc1nccc(-c2cc(-c3ccc(Cl)cc3Cl)no2)n1)Nc1ccc(C(F)(F)F)cc1,15000
Cc1nc(SCC(=O)Nc2ccc([N+](=O)[O-])cc2)nc(O)c1C,1000000
CC(C)(C)c1ccc(NC(=O)CSc2nc(O)c(C#N)c(C3CCCCC3)n2)cc1,350000
NC(=O)c1ccc2c(c1)C(=O)C(=O)N2Cc1ccccc1,12500
O=C1C(=O)N(Cc2ccc3ccccc3c2)c2ccc(I)cc21,1100
COc1ccc(NC(=O)CSc2nc(O)cc(-c3ccccc3)n2)cc1OC,45000
CCOc1ccc(N2C(=O)CC(Sc3nc(C)cc(C)n3)C2=O)cc1,3311311214825.91
CC(=O)c1ccccc1S(=O)(=O)c1ccccc1C(=O)O,16000
CC1(C)CC(=O)c2c(NCc3ccco3)sc(C#N)c2C1,16000
Cc1cc(O)nc(SCC(=O)Nc2cc(Cl)ccc2Oc2ccccc2)n1,100000
CCCCc1ccc(NC(=O)CSc2nc(O)c(C#N)c(C3CCCCC3)n2)c(C)c1,200000
CC(C)C1=Cc2ccc3c(c2C(=O)C1=O)CCCC3(C)C,78900
O=C(Oc1ccc(S(=O)(=O)c2ccc(OC(=O)C(Cl)=C(Cl)Cl)cc2)cc1)C(Cl)=C(Cl)Cl,900
NC(=O)c1ccc2c(c1)C(=O)C(=O)N2Cc1ccc2ccccc2c1,370
CCCN1C(=O)C(=O)c2cc(C(N)=O)ccc21,25000
O=C1C(=O)N(Cc2ccccc2)c2ccc(I)cc21,50000
Cc1cccc2c3c(ccc12)C1=C(C(=O)C3=O)[C@@H](C)CO1,4900
COC(=O)[C@@]1(C)CCCc2c1ccc1c2C(=O)C(=O)c2c(C)coc2-1,21100
O=C1C(=O)N(C/C=C/c2cc3ccccc3s2)c2ccc(I)cc21,23500
O=C(CSc1nccc(-c2csc(-c3ccccc3)n2)n1)Nc1cc(Cl)cc(Cl)c1,331131121482591
CC(C)c1ccc(NC(=O)CSc2nccc(-c3cccs3)n2)cc1,40000
Cc1cc(C(F)(F)F)nc(SCC(=O)Nc2ccc(Cl)cc2F)n1,1995262314968.88
COc1ccc(-c2ccnc(SCC(=O)Nc3ccc(C(C)C)cc3)n2)cc1,400000
CC(Sc1nc(O)c(C#N)c(-c2ccccc2)n1)C(=O)Nc1ccc(Cl)cc1,3311311214825.91
N#Cc1c(O)nc(SCC(=O)Nc2cccc(C(F)(F)F)c2)nc1-c1ccccc1,200000
CC(C)c1ccc(NC(=O)CSc2nccc(-c3csc(COc4ccccc4Cl)n3)n2)cc1,4897788193684.46
COc1cccc(-c2nc(SCC(=O)Nc3ccc(S(N)(=O)=O)cc3)nc(O)c2C#N)c1,40000
Cc1coc2c1C(=O)C(=O)c1c-2ccc2c1CCCC2(C)CO,10700
CC(C)C1=Cc2ccc3c(c2C(=O)C1=O)CCCC3(C)C,21100
Cc1coc2c1C(=O)C(=O)c1c-2ccc2c(C)cccc12,700
Cc1coc2c1C(=O)C(=O)c1c-2ccc2c(C)cccc12,11500
CC(C)C1=Cc2ccc3c(c2C(=O)C1=O)CCCC3(C)C,60200
Cc1coc2c1C(=O)C(=O)c1c-2ccc2c1CCCC2(C)C,17100
Cc1cccc2c3c(ccc12)C1=C(C(=O)C3=O)[C@@H](C)CO1,10700
Cc1coc2c1C(=O)C(=O)c1c-2ccc2c1CCCC2(C)CO,18000
COC(=O)[C@@]1(C)CCCc2c1ccc1c2C(=O)C(=O)c2c(C)coc2-1,10600
Cc1coc2c1C(=O)C(=O)c1c-2ccc2c(C)cccc12,8800
Cc1coc2c1C(=O)C(=O)c1c-2ccc2c1CCCC2(C)CO,24800

COC(=O)[C@@]1(C)CCCc2c1ccc1c2C(=O)C(=O)c2c(C)coc2-1,9200

Compounds with any activity data on viral cysteine proteases were extracted from ChEMBL (4.11.2020) and the complete file in csv format is supplied as part of supporting information. The decoyes employed were obtained as a standard 1k decoy set from Schrödinger LLC.

chembl_id,canonical_smiles,organism,full_mwt,l1,l2,l3

3.1 Machine Learning

Herein, the following steps are of relevance. First, the database of relevant target and decoy molecules is constructed. Next, stratified splits of the data are obtained – these types of data splits ensure approximately equal distributions of different classes (relevant for classification). Multiple splits are obtained, and for each one, a model is trained and evaluated. As the best performing model was a neural network, we emphasize this model next. The overall architecture can be summarized as follows:

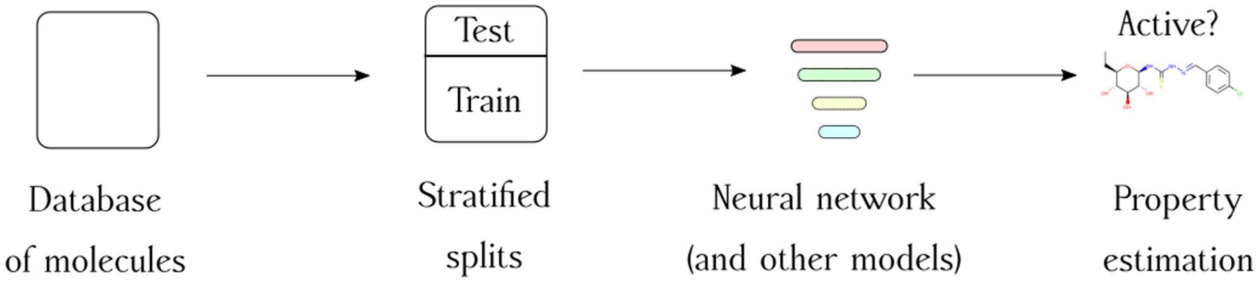

Figure S3: The overview of the learning process.

The architecture can be understood as a feedforward neural network with additional dropout layers attached. The final layer is either a ReLU – in this case the network performs regression for one target, or sigmoid, in which case it performs binary classification. Feature space was constructed as follows. We used the DeepChem library to produce RDKit descriptors via the "RDKitDescriptors()" call on the input SMILES-formatted molecules. The considered descriptors were the following ones and referenced in the section RDKit (https://www.rdkit.org/docs/GettingStartedInPython.html#list-of-available-descriptors):

['MaxEStateIndex', 'MinEStateIndex', 'MaxAbsEStateIndex', 'MinAbsEStateIndex', 'qed', 'MolWt', 'HeavyAtomMolWt', 'ExactMolWt', 'NumValenceElectrons', 'NumRadicalElectrons',

'MaxPartialCharge', 'MinPartialCharge', 'MaxAbsPartialCharge', 'MinAbsPartialCharge', 'FpDensityMorgan1', 'FpDensityMorgan2', 'FpDensityMorgan3', 'BCUT2D_MWHI', 'BCUT2D_MWLOW', 'BCUT2D_CHGHI', 'BCUT2D_CHGLO', 'BCUT2D_LOGPHI', 'BCUT2D_LOGPLOW', 'BCUT2D_MRHI', 'BCUT2D_MRLOW', 'BalabanJ', 'BertzCT', 'Chi0', 'Chi0n', 'Chi0v', 'Chi1', 'Chi1n', 'Chi1v', 'Chi2n', 'Chi2v', 'Chi3n', 'Chi3v', 'Chi4n', 'Chi4v', 'HallKierAlpha', 'Ipc', 'Kappa1', 'Kappa2', 'Kappa3', 'LabuteASA', 'PEOE_VSA1', 'PEOE_VSA10', 'PEOE_VSA11', 'PEOE_VSA12', 'PEOE_VSA13', 'PEOE_VSA14', 'PEOE_VSA2', 'PEOE_VSA3', 'PEOE_VSA4', 'PEOE_VSA5', 'PEOE_VSA6', 'PEOE_VSA7', 'PEOE_VSA8', 'PEOE_VSA9', 'SMR_VSA1', 'SMR_VSA10', 'SMR_VSA2', 'SMR_VSA3', 'SMR_VSA4', 'SMR_VSA5', 'SMR_VSA6', 'SMR_VSA7', 'SMR_VSA8', 'SMR_VSA9', 'SlogP_VSA1', 'SlogP_VSA10', 'SlogP_VSA11', 'SlogP_VSA12', 'SlogP_VSA2', 'SlogP_VSA3', 'SlogP_VSA4', 'SlogP_VSA5', 'SlogP_VSA6', 'SlogP_VSA7', 'SlogP_VSA8', 'SlogP_VSA9', 'TPSA', 'EState_VSA1', 'EState_VSA10', 'EState_VSA11', 'EState_VSA2', 'EState_VSA3', 'EState_VSA4', 'EState_VSA5', 'EState_VSA6', 'EState_VSA7', 'EState_VSA8', 'EState_VSA9', 'VSA_EState1', 'VSA_EState10', 'VSA_EState2', 'VSA_EState3', 'VSA_EState4', 'VSA_EState5', 'VSA_EState6', 'VSA_EState7', 'VSA_EState8', 'VSA_EState9', 'FractionCSP3', 'HeavyAtomCount', 'NHOHCount', 'NOCount', 'NumAliphaticCarbocycles', 'NumAliphaticHeterocycles', 'NumAliphaticRings', 'NumAromaticCarbocycles', 'NumAromaticHeterocycles', 'NumAromaticRings', 'NumHAcceptors', 'NumHDonors', 'NumHeteroatoms', 'NumRotatableBonds', 'NumSaturatedCarbocycles', 'NumSaturatedHeterocycles', 'NumSaturatedRings', 'RingCount', 'MolLogP', 'MolMR', 'fr_Al_COO', 'fr_Al_OH', 'fr_Al_OH_noTert', 'fr_ArN', 'fr_Ar_COO', 'fr_Ar_N', 'fr_Ar_NH', 'fr_Ar_OH', 'fr_COO', 'fr_COO2', 'fr_C_O', 'fr_C_O_noCOO', 'fr_C_S', 'fr_HOCCN', 'fr_Imine', 'fr_NH0', 'fr_NH1', 'fr_NH2', 'fr_N_O', 'fr_Ndealkylation1', 'fr_Ndealkylation2', 'fr_Nhpyrrole', 'fr_SH', 'fr_aldehyde', 'fr_alkyl_carbamate', 'fr_alkyl_halide', 'fr_allylic_oxid', 'fr_amide', 'fr_amidine', 'fr_aniline', 'fr_aryl_methyl', 'fr_azide', 'fr_azo', 'fr_barbitur', 'fr_benzene', 'fr_benzodiazepine', 'fr_bicyclic', 'fr_diazo', 'fr_dihydropyridine', 'fr_epoxide', 'fr_ester', 'fr_ether', 'fr_furan', 'fr_guanido', 'fr_halogen', 'fr_hdrzine', 'fr_hdrzone', 'fr_imidazole', 'fr_imide', 'fr_isocyan', 'fr_isothiocyan', 'fr_ketone', 'fr_ketone_Topliss', 'fr_lactam', 'fr_lactone', 'fr_methoxy', 'fr_morpholine', 'fr_nitrile', 'fr_nitro', 'fr_nitro_arom', 'fr_nitro_arom_nonortho', 'fr_nitroso', 'fr_oxazole', 'fr_oxime', 'fr_para_hydroxylation', 'fr_phenol', 'fr_phenol_noOrthoHbond', 'fr_phos_acid', 'fr_phos_ester', 'fr_piperdine', 'fr_piperzine', 'fr_priamide', 'fr_prisulfonamd', 'fr_pyridine', 'fr_quatN', 'fr_sulfide', 'fr_sulfonamd', 'fr_sulfone', 'fr_term_acetylene', 'fr_tetrazole', 'fr_thiazole', 'fr_thiocyan', 'fr_thiophene', 'fr_unbrch_alkane', 'fr_urea']

The descriptors are also referenced and described further in the Supporting Information under the section RDKit.

5. Physicochemical properties

Compound descriptors were calculated using QikProp by Schrödinger and RDKit (rdkit.org; https://www.rdkit.org/docs/index.html; accessed on 10.1.2021). [63]

QikProp:

- molecule name

Molecule name taken from the title line in the input structure file. If the title line is blank, the input file name is used.

- #stars

Number of property or descriptor values that fall outside the 95% range of similar values for known drugs. Outlying descriptors and predicted properties are denoted with asterisks (*) in the .out file. A large number of stars suggests that a molecule is less drug-like than molecules with few stars. The following properties and descriptors are included in the determination of #stars: MW, dipole, IP, EA, SASA, FOSA, FISA, PISA, WPSA, PSA, volume, #rotor, donorHB, accptHB, glob, QPpolrz, QPlogPC16, QPlogPoct, QPlogPw, QPlogPo/w, logS, QPLogKhsa, QPlogBB, #metabol
0 – 5

- #amine

Number of non-conjugated amine groups.
0 – 1

- #amidine

Number of amidine and guanidine groups.
0

- #acid

Number of carboxylic acid groups.
0 – 1

- #amide

Number of non-conjugated amide groups.
0 – 1

- #rotor

Number of non-trivial (not CX3), non-hindered (not alkene, amide, small ring) rotatable bonds.
0 – 15

- #rtvFG

Number of reactive functional groups; the specific groups are listed in the jobname .out file. The presence of these groups can lead to false positives in HTS assays and to decomposition, reactivity, or toxicity problems in vivo. See Appendix A of the QikProp User Manual for a complete list.
0 – 2

- CNS

Predicted central nervous system activity on a –2 (inactive) to +2 (active) scale.
–2 to +2

- mol_MW

Molecular weight of the molecule.
130.0 – 725.0

- dipole

Computed dipole moment of the molecule.
1.0 – 12.5

- SASA

Total solvent accessible surface area (SASA) in square angstroms using a probe with a 1.4 Å radius.
300.0 – 1000.0

- FOSA

Hydrophobic component of the SASA (saturated carbon and attached hydrogen).
0.0 – 750.0

- FISA

Hydrophilic component of the SASA (SASA on N, O, H on heteroatoms, and carbonyl C).
7.0 – 330.0

- PISA

π (carbon and attached hydrogen) component of the SASA.
0.0 – 450.0

- WPSA

Weakly polar component of the SASA (halogens, P, and S).
0.0 – 175.0

- volume

Total solvent-accessible volume in cubic angstroms using a probe with a 1.4 Å radius.
500.0 – 2000.0

- donorHB

Estimated number of hydrogen bonds that would be donated by the solute to water molecules in an aqueous solution. Values are averages taken over a number of configurations, so they can be non-integer.
0.0 – 6.0

- accptHB

Estimated number of hydrogen bonds that would be accepted by the solute from water molecules in an aqueous solution. Values are averages taken over a number of configurations, so they can be non-integer.
2.0 – 20.0

- dip^2/V

Square of the dipole moment divided by the molecular volume. This is the key term in the Kirkwood-Onsager equation for the free energy of solvation of a dipole with volume V.
0.0 – 0.13

- ACxDN^.5/SA

Index of cohesive interaction in solids. This term represents the relationship
; see Bioorg. Med. Chem. Lett. 2000, 10, 1155.
0.0 – 0.05

- glob

Globularity descriptor,
, where r is the radius of a sphere with a volume
equal to the molecular volume. Globularity is 1.0 for a spherical molecule.
0.75 – 0.95

- QPpolrz

Predicted polarizability in cubic angstroms.
13.0 – 70.0

- QPlogPC16

Predicted hexadecane/gas partition coefficient.
4.0 – 18.0

- QPlogPoct

Predicted octanol/gas partition coefficient.
8.0 – 35.0

- QPlogPw

Predicted water/gas partition coefficient.
4.0 – 45.0

- QPlogPo/w

Predicted octanol/water partition coefficient.
–2.0 – 6.5

- QPlogS

Predicted aqueous solubility, log S. S in mol dm–3 is the concentration of the solute in a saturated solution that is in equilibrium with the crystalline solid.
–6.5 – 0.5

- CIQPlogS

Conformation-independent predicted aqueous solubility, log S. S in mol dm–3 is the concentration of the solute in a saturated solution that is in equilibrium with the crystalline solid.
–6.5 – 0.5

- QPlogHERG

Predicted IC50 value for blockage of HERG K+ channels.
concern below –5

- QPPCaco

Predicted apparent Caco-2 cell permeability in nm/sec. Caco-2 cells are a model for the gut-blood barrier. QikProp predictions are for non-active transport.
<25 poor,

>500 great

- QPlogBB

Predicted brain/blood partition coefficient. Note: QikProp predictions are for orally delivered drugs so, for example, dopamine and serotonin are CNS negative because they are too polar to cross the blood-brain barrier
–3.0 – 1.2

- QPPMDCK

Predicted apparent MDCK cell permeability in nm/sec. MDCK cells are considered to be a good mimic for the blood-brain barrier. QikProp predictions are for non-active transport.
<25 poor,
>500 great

- QPlogKp

Predicted skin permeability, log Kp.
–8.0 – –1.0

- IP(ev)

PM3 calculated ionization potential (negative of HOMO energy).
7.9 – 10.5

- EA(eV)

PM3 calculated electron affinity (negative of LUMO energy).
–0.9 – 1.7

- #metab

Number of likely metabolic reactions. See Appendix A of the QikProp User Manual for a complete list of reactions.
1 – 8

- QPlogKhsa

Prediction of binding to human serum albumin.
–1.5 – 1.5

- HumanOralAbsorption

Predicted qualitative human oral absorption: 1, 2, or 3 for low, medium, or high. The text version is reported in the output. The assessment uses a knowledge-based set of rules, including checking for suitable values of PercentHumanOralAbsorption, number of metabolites, number of rotatable bonds, logP, solubility and cell permeability.

- PercentHumanOralAbsorption

Predicted human oral absorption on 0 to 100% scale.The prediction is based on a quantitative multiple linear regression model. This property usually correlates well with HumanOralAbsorption, as both measure the same property.
>80% is high
<25% is poor

- SAFluorine

Solvent-accessible surface area of fluorine atoms.
0.0 – 100.0

- SAamideO

Solvent-accessible surface area of amide oxygen atoms.
0.0 – 35.0

- PSA

Van der Waals surface area of polar nitrogen and oxygen atoms and carbonyl carbon atoms.
7.0 – 200.0

- #NandO

Number of nitrogen and oxygen atoms.
2 – 15

- RuleOfFive

Number of violations of Lipinski's rule of five. The rules are: mol_MW < 500, QPlogPo/w < 5, donorHB ≤ 5, accptHB ≤ 10. Compunds that satisfy these rules are considered drug-like. (The "five" refers to the limits, which are multiples of 5.)
maximum is 4

- RuleOfThree

Number of violations of Jorgensen's rule of three. The three rules are: , QPlogS > -5.7, QP PCaco > 22 nm/s, # Primary Metabolites < 7. Compounds with fewer (and preferably no) violations of these rules are more likely to be orally available.
maximum is 3

- #ringatoms

Number of atoms in rings.

- #in34

Number of atoms in 3- or 4-membered rings.

- #in56

Number of atoms in 5- or 6-membered rings.

- #noncon

number of ring atoms not able to form conjugated aromatic systems (e.g. sp3 C).

- #nonHatm

Number of heavy atoms (nonhydrogen atoms).

- Jm

Predicted maximum transdermal transport rate, Kp × MW × S (μg cm–2 hr–1). Kp and S are obtained from the aqueous solubility and skin permeability, QPlogKp and QPlogS. This property is only written to the output file: it is not used in any other calculations.

RDKit:

List of Available (2D) Descriptors as in v. 2020.09.1:

Descriptor/Descriptor Family	Notes
Gasteiger/Marsili Partial Charges	*Tetrahedron* **36**:3219-28 (1980)
BalabanJ	*Chem. Phys. Lett.* **89**:399-404 (1982)
BertzCT	*J. Am. Chem. Soc.* **103**:3599-601 (1981)
Ipc	*J. Chem. Phys.* **67**:4517-33 (1977)
HallKierAlpha	*Rev. Comput. Chem.* **2**:367-422 (1991)
Kappa1 - Kappa3	*Rev. Comput. Chem.* **2**:367-422 (1991)
Chi0, Chi1	*Rev. Comput. Chem.* **2**:367-422 (1991)
Chi0n - Chi4n	*Rev. Comput. Chem.* **2**:367-422 (1991)
Chi0v - Chi4v	*Rev. Comput. Chem.* **2**:367-422 (1991)
MolLogP	Wildman and Crippen *JCICS* **39**:868-73 (1999)
MolMR	Wildman and Crippen *JCICS* **39**:868-73 (1999)
MolWt	
ExactMolWt	
HeavyAtomCount	
HeavyAtomMolWt	
NHOHCount	
NOCount	
NumHAcceptors	
NumHDonors	
NumHeteroatoms	
NumRotatableBonds	
NumValenceElectrons	
NumAmideBonds	
Num{Aromatic,Saturated,Aliphatic}Rings	
Num{Aromatic,Saturated,Aliphatic}{Hetero,Carbo}cycles	
RingCount	
FractionCSP3	
NumSpiroAtoms	Number of spiro atoms (atoms shared between rings that share exactly one atom)
NumBridgeheadAtoms	Number of bridgehead atoms (atoms shared between rings that share at least two bonds)
TPSA	*J. Med. Chem.* **43**:3714-7, (2000) See the section in the RDKit book describing differences to the original publication.
LabuteASA	*J. Mol. Graph. Mod.* **18**:464-77 (2000)
PEOE_VSA1 - PEOE_VSA14	MOE-type descriptors using partial charges and surface area

	contributions http://www.chemcomp.com/journal/vsadesc.htm
SMR_VSA1 - SMR_VSA10	MOE-type descriptors using MR contributions and surface area contributions http://www.chemcomp.com/journal/vsadesc.htm
SlogP_VSA1 - SlogP_VSA12	MOE-type descriptors using LogP contributions and surface area contributions http://www.chemcomp.com/journal/vsadesc.htm
EState_VSA1 - EState_VSA11	MOE-type descriptors using EState indices and surface area contributions (developed at RD, not described in the CCG paper)
VSA_EState1 - VSA_EState10	MOE-type descriptors using EState indices and surface area contributions (developed at RD, not described in the CCG paper)
MQNs	Nguyen et al. *ChemMedChem* **4**:1803-5 (2009)
Topliss fragments	implemented using a set of SMARTS definitions in $(RDBASE)/Data/FragmentDescriptors.csv
Autocorr2D	New in 2017.09 release. Todeschini and Consoni "Descriptors from Molecular Geometry" Handbook of Chemoinformatics https://doi.org/10.1002/9783527618279.ch37
BCUT2D	New in 2020.09 release. Pearlman and Smith in "3D-QSAR and Drug design: Recent Advances" (1997)

SUPPORTING INFORMATION REFERENCES

[62] Jukic, M. *et al*. CmDock, *https://gitlab.com/Jukic/cmdock/*; **2021**.

[63] Tosco, P., Stiefl, N., & Landrum, G. (2014). Bringing the MMFF force field to the RDKit: implementation and validation. *Journal of Cheminformatics*, *6*(1), 1-4.
